# Extracellular cGAMP in health and disease

**DOI:** 10.1186/s43556-026-00464-x

**Published:** 2026-05-10

**Authors:** Zhongling Dai, Chenggong Ma, Huiqing Ding, Liyao Fu, Hejun Jiang, Shi Tai

**Affiliations:** 1 Department of Cardiology, The Second Xiangya Hospital of Central South University, Changsha, 410011 China; 2 Department of Blood Transfusion, The Second Xiangya Hospital of Central South University, Changsha, China

**Keywords:** CGAMP, Infection, Tumor, Aging, Tissue homeostasis, Therapeutic strategies

## Abstract

The cGAS-STING signaling pathway is a crucial component of the innate immune system that detects aberrant cytosolic DNA, such as that derived from viruses or damaged cells, to activate downstream immune responses. Within this pathway, cyclic guanosine monophosphate–adenosine monophosphate (cGAMP) serves as the essential second messenger linking DNA sensing to immune activation. Upon recognition of cytosolic DNA, cGAS synthesizes cGAMP, whose unique "mixed linkage" structure enables efficient binding to and activation of the STING protein on the endoplasmic reticulum, thereby inducing type I interferons and inflammatory cytokines. This review details cGAMP’s biosynthesis, structural characteristics, and transport mechanisms, including efflux via ABCC1 and uptake by SLC19A1, underscoring its role as an intercellular "immune messenger." It also explores the dual functions of cGAMP in antiviral and antitumor immunity as well as in autoimmune and aging-related diseases, where it can either enhance immune defense or promote chronic inflammation. Therapeutically, cGAMP has been investigated as a vaccine adjuvant, a target for synthesis or degradation enzymes, and in nanoparticle-based delivery systems. However, challenges regarding its stability, delivery efficiency, and immunotoxicity remain, and future research should focus on real-time monitoring and tissue-specific modulation to advance cGAMP-based precision immunotherapeutics.

## Introduction

Cyclic guanosine monophosphate (GMP)-adenosine monophosphate (AMP) (cGAMP), a dimeric cyclic nucleotide synthesized by the intracellular enzyme cyclic GMP-AMP synthase (cGAS), functions as a crucial second messenger in the signalling pathway of cGAS-stimulator of interferon genes (STING). This pathway senses cytosolic DNA and is essential for initiating innate immune responses. Upon detecting exogenous DNA from pathogens or endogenous DNA leaked from damaged organelles in the cytosol, cGAS catalyzes the conversion of ATP and GTP into cGAMP, which subsequently engages with STING to initiate downstream signalling cascades. Activated STING further activates TANK-binding kinase 1 (TBK1), leading to the phosphorylation and activation of interferon regulatory factor 3 (IRF3) and nuclear factor kappa-light-chain-enhancer of activated B cells (NF-κB), ultimately inducing the expression of Type I interferons (IFN) and inflammatory cytokines [1–5]. Type I IFN exhibit diverse immunomodulatory properties, including anti-infection, antitumor, and senescence-inducing effects [6–11]. Emerging evidence has also highlighted the cGAS-STING pathway as a key regulator of the senescence-associated secretory phenotype (SASP), making it a target in aging therapeutics. The release of self-DNA into the cytoplasm of senescent cells activates the cGAS-STING-NF-κB signalling pathway via this signalling route [12–15], involving the secretion of SASP [16–20], which may contribute to immune dysregulation and chronic inflammation in the context of aging. Thus, the cGAS-cGAMP-STING axis represents a fundamental mechanism linking intracellular DNA sensing to systematic immune and inflammatory outcomes.

Recent investigations have revealed that cGAMP operates not only intracellularly, but also as an “immune messenger” that translocates between cells via specific transport proteins, thus facilitating precisely orchestrated intercellular communication [4, 15, 21]. Through this mechanism, cGAMP potentiates antitumor defenses and stimulates pro-inflammatory responses to facilitate pathogen clearance [21, 22]. Despite growing interest in cGAMP-mediated immune surveillance, antiviral responses, and antitumor immunity, its role in age-related immune responses remains poorly understood. Through a comprehensive understanding of intercellular cGAMP signalling, we could elucidate dynamic alterations in tissue microenvironments and interactions between diseased and healthy cells, particularly in the context of aging.

This review aims to systematically examine the multifaceted roles of cGAMP, from its molecular synthesis to its complex functions in physiology and pathology, with a particular focus on its emerging significance in aging. We first described the molecular mechanisms of cGAMP biosynthesis and its unique structural features. Next, we discussed cGAMP transport mechanisms, including efflux via ABCC1 and uptake by SLC19A1, emphasizing its function as an intercellular immune messenger. We then explored the dual roles of cGAMP in antiviral and antitumor immunity, as well as its contributions to autoimmune and aging-related diseases, where it could either enhance immune defense or promote chronic inflammation. Finally, we addressed therapeutic applications of cGAMP, including its use as a vaccine adjuvant, modulation of enzymes governing its synthesis and degradation, and nanoparticle-based delivery systems, and discussed current challenges and future directions for precision immunomodulation.

## Biosynthesis and molecular identity Of cGAMP

To fully appreciate diverse functions of cGAMP, particularly its capacity to function as both an intracellular second messenger and an intercellular immune signal, it is essential to first understand its origins and distinctive molecular characteristics. The unique structural features of cGAMP, most notably its mixed 2’−5’/3’−5’ phosphodiester linkage, underpin its high-affinity binding to STING and its resistance to certain degradation pathways. These properties distinguish it from bacterial cyclic dinucleotides and enable its specialized role in mammalian immunity. Therefore, this section examines cGAS structure, dsDNA activation mechanisms, the catalytic mechanism of cGAMP synthesis, and the structural determinants that confer upon cGAMP its potent immunostimulatory activity.

### cGAS structure and dsDNA activation

cGAS is a nucleotidyltransferase comprising an N-terminal regulatory region and a C-terminal catalytic domain [23–25]. In the absence of DNA, the enzyme adopts an autoinhibitory conformation wherein the N-terminal domain sterically occludes the catalytic pocket, maintaining basal enzymatic activity at minimal levels [25–27]. Recognition of double-stranded DNA (dsDNA) occurs through three positively charged DNA-binding surfaces that engage the sugar-phosphate backbone via non-sequence-specific electrostatic and hydrogen bonding interactions [28, 29]. Due to this binding mode, cGAS can respond to dsDNA from diverse sources—viral, bacterial, or host-derived (e.g., from damaged or tumor cells)—without requiring specific nucleotide sequence recognition.

Upon DNA binding, two cGAS molecules assemble into a 2:2 stoichiometric complex oriented in a “back-to-back” configuration [30–32]. This dimerization event induces substantial conformational rearrangements, including the displacement of the N-terminal inhibitory domain and exposure of the catalytic pocket and substrate access channel. Notably, cGAS-dsDNA complexes undergo liquid–liquid phase separation (LLPS), forming biomolecular condensates that concentrate multiple cGAS molecules along the DNA lattice [33]. This phase separation generates a specialized microenvironment that dramatically enhances catalytic efficiency by increasing local enzyme concentration and facilitating substrate channeling. The LLPS-mediated assembly represents a critical regulatory mechanism that enables robust signal amplification from limited cytosolic DNA stimuli.

### Catalytic mechanism of cGAS

**Fig. 1:**
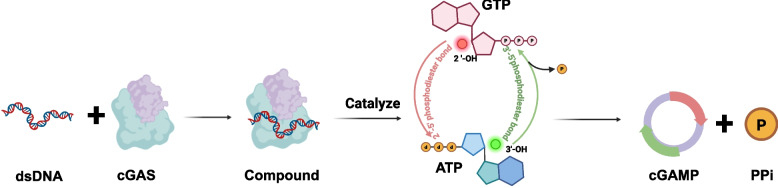
Synthesis of cGAMP. cGAS recognizes DNA and then facilitates a nucleophilic attack where the 2′-hydroxyl group of the guanine ribose (from GTP) on the α-phosphate of the ATP molecule, resulting in a 2′−5′ phosphodiester bond and yielding the linear intermediate 5′-ppG(2′−5′)A. Concurrently, the 3′-hydroxyl group of the adenine ribose (from ATP) attacks the α-phosphate of the initiating GTP molecule, forming a 3′−5′ phosphodiester bond. This integrated, concerted mechanism culminates in cyclization, producing the unique cyclic dinucleotide product, 2′3′-cGAMP (G(2′−5′)pA(3′−5′)p), and releasing an inorganic pyrophosphate (PPi) molecule as a byproduct

The C-terminal catalytic domain of cGAS simultaneously binds ATP and GTP substrates in a bifurcated nucleotide-binding pocket [25, 30, 34, 35]. The synthesis of 2′3’-cGAMP proceeds through a two-step nucleotidyl transfer reaction with strict stereochemical control (Fig. 1). The process initiates with the formation of a stable cGAS-dsDNA complex, where dsDNA acts as an allosteric activator. This binding event triggers a nucleotide transfer reaction [36–39]: cGAS facilitates a nucleophilic attack by the 2’-hydroxyl group of the guanine ribose (from GTP) on the α-phosphate of the ATP molecule. This results in a 2’−5’ phosphodiester bond, yielding the linear intermediate 5′-ppG(2’−5’)A. In a subsequent, seemingly concerted step, the 3’-hydroxyl group of the adenine ribose (from the same ATP molecule) attacks the α-phosphate of the initiating GTP molecule, forming a 3’−5’ phosphodiester bond. In this cyclization step, 2′3’-cGAMP (G(2’−5’)pA(3’−5’)p), the final, unique cyclic dinucleotide product, is produced, and an inorganic pyrophosphate (PPi) molecule is released as a byproduct (Fig. 1). The catalytic mechanism distinguishes cGAS from other nucleotidyltransferases and ensures the exclusive production of the 2′3’-cGAMP isomer, which exhibits optimal STING-binding properties.

### Unique structural properties of cGAMP

The functional uniqueness of cGAMP is its non-canonical phosphodiester linkage topology, which fundamentally differs from bacterial cyclic dinucleotides (CDNs). Bacterial cyclic dinucleotides, such as c-di-GMP and c-di-AMP, typically exhibit symmetrical structures interconnected solely by two standard 3’−5’ phosphodiester bonds [40–42]. In stark contrast, 2′3’-cGAMP possesses a unique “mixed linkage” architecture, incorporating both a 2’−5’ and a 3’−5’ phosphodiester bond in a single molecule [5, 43–45].

This specific configuration is critically important for high-affinity binding to its receptor. The distinct geometry of 2′3’-cGAMP allows it to fit perfectly in the V-shaped binding pocket formed at the dimer interface of the STING protein [46–51]. This high-affinity interaction induces a dramatic conformational transition of STING from an “open” to a “closed” active state, initiating downstream oligomerization, translocation from the endoplasmic reticulum, and phosphorylation cascades that culminate in Type I interferon production. Notably, 2′3’-cGAMP exhibits markedly higher binding affinity for human STING compared to bacterial 3’−3’ linked CDNs [52, 53]. This superior binding affinity translates into more potent and sustained pathway activation, so that minimal cytosolic DNA signals can elicit robust interferon responses. Consequently, 2′3’-cGAMP functions as an efficient “self-danger signal amplifier,” ensuring that mammalian cells preferentially respond to endogenous cGAMP over structurally similar bacterial molecules. Thus, the structural determinants of cGAMP not only establish the molecular basis for its role as a highly specific and sensitive immunotransmitter but also set the stage for understanding how this molecule traverses cellular boundaries to coordinate immune responses across tissues—a subject we address in the following section.

## The transport mechanism Of cGAMP

The distinctive molecular architecture of cGAMP, particularly its hydrophilic and negatively charged nature, presents a fundamental paradox: How can a molecule that cannot passively diffuse across lipid bilayers nevertheless function as an intercellular signal? The resolution to this paradox is the evolution of specialized transport systems that enable cGAMP to traverse cellular boundaries and coordinate multicellular immune responses. Building upon our understanding of cGAMP synthesis and structure, this section examines the mechanisms governing cGAMP export from producer cells, its extracellular stability, uptake by recipient cells, and the functional consequences of intercellular cGAMP transfer in diverse physiological contexts. These transport mechanisms are not merely logistical details. They fundamentally shape the spatiotemporal dynamics of cGAMP signalling and determine whether its effects remain localized or propagate systemically.

### Extracellular stability and degradation Of cGAMP

**Fig. 2:**
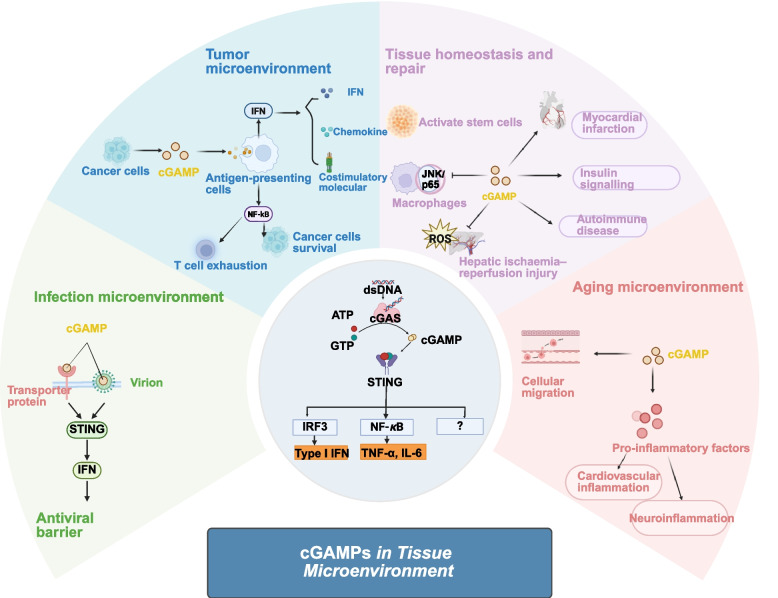
Intercellular transport mechanism of cGAMP. As a pivotal intracellular signaling molecule, cGAMP is synthesized by the enzyme cGAS. It facilitates intercellular communication through various transporters or specialized channelsthereby triggering immune responses associated with cellular senescence

The biological activity of extracellular cGAMP is constrained by its rapid enzymatic degradation. Two major classes of extracellular cGAMP hydrolases have been identified: ectonucleotide pyrophosphatase/phosphodiesterase family members (ENPP1, ENPP3) [54, 55] and sphingomyelin phosphodiesterase-like 3 A (SMPDL3A) [56]. These enzymes efficiently hydrolyze 2′3’-cGAMP, limiting its extracellular diffusion and half-life. ENPP1 and ENPP3 share identical domain arrangements and both utilize conserved residues, such as Thr238, to carry out nucleophilic attacks on the 2’−5’ and 3’−5’ phosphodiester linkages of cGAMP, ultimately hydrolyzing it into AMP and GMP [54, 57]. However, ENPP3 exhibits lower catalytic efficiency due to significant accumulation of reaction intermediates [54, 58]. In contrast, SMPDL3A functions as a cGAMP‑specific nuclease. It cleaves the 2’−5’ and 3’−5’ phosphodiester bonds via a substrate‑induced dimerization mechanism, thus accelerating the clearance of extracellular cGAMP [56, 59]. Two principal mechanisms protect cGAMP from premature degradation and enable its intercellular transmission [21]: The first involves its movement through gap junctions, which circumvents exposure to extracellular hydrolases; and the second pertains to carrier-mediated transport, including encapsulation by transport proteins and membrane vesicles (Fig. 2). This intricate balance between synthesis, degradation, and protected transfer dictates the spatial range and temporal mics of cGAMP signalling.

### Output and efflux pathways of cGAMP

As a hydrophilic and negatively charged molecule, cGAMP cannot passively cross the plasma membrane [60–62]. Therefore, its export from cells relies on specialized mechanisms, which can be broadly categorized as passive release, active transport, or membrane-encapsulated release [30, 60]. Active transport is the primary pathway for regulated cGAMP export. ATP-binding Cassette Family C Member 1 (ABCC1, also known as MRP1) is the most extensively characterized transporter. ABCC1 possesses a 17-transmembrane helix topology organized into two nucleotide-binding domains (NBD1 and NBD2) and two transmembrane domains that form the substrate translocation pathway. This architecture enables ATP-dependent substrate transport: cGAMP binding to the inward-facing cavity induces conformational changes that bring NBD1 and NBD2 into proximity for ATP binding [63–65]. Subsequent ATP hydrolysis drives transition to an outward-facing conformation, opening the extracellular gate and actively extruding cGAMP [63, 64]. Thus, the 17-transmembrane architecture provides both the structural scaffold for substrate recognition and the coordinated domain movements essential for vectorial transport. This export mechanism serves a dual physiological purpose: It not only regulates the extracellular concentration of cGAMP available for intercellular signalling, but also attenuates the intensity of STING signalling in the producing cell itself, thus preventing excessive immune activation. Importantly, the conformational coupling between substrate binding and ATP hydrolysis ensures that cGAMP export is tightly coupled to cellular energy status, providing a metabolic checkpoint for immune signal propagation. Additionally, in the most recent study, ABCC10 (MRP7) has been identified as another efflux transporter capable of exporting cGAMP, a process linked to cancer cell resistance to ionizing radiation [66]. This discovery of a second cGAMP exporter raises intriguing questions about functional redundancy and specialization: Whether ABCC1 and ABCC10 operate in distinct cellular contexts, respond to different stimuli, or exhibit substrate preferences that fine-tune cGAMP availability in specific microenvironments requires further investigation. By altering these transport proteins or anion channels to enhance cGAMP transfer to adjacent cells, STING-mediated immune responses can be potentiated [67]. Although proteins associated with the extracellular release of cGAMP have been identified, the exact mechanisms regulating its exit from the cell remain unclear. One possible mechanism involves the passive release of cGAMP from damaged cell membranes during cell death. Alternatively, another hypothesis suggests an intracellular maturation process for cGAMP trafficking, namely membrane-encapsulated release. The passive release mechanism mainly occurs under conditions of compromised plasma membrane integrity, representing a non-specific “leakage”. Substances, including cGAMP, are passively released into the extracellular space [1, 21, 68]. It can subsequently be taken up by adjacent healthy cells with intact membranes, such as dendritic cells and macrophages, hereby amplifying the immune response. While it is rapid and uncontrolled, this process can act as a potent danger signal, alerting adjacent healthy cells (Fig. 2).

Furthermore, cGAMP can be packaged into and released via membrane-enclosed vesicles, enabling long-distance intercellular signalling and protection from extracellular degradation. Studies have revealed that tumor cells can transfer cGAMP to immune cells in extracellular vesicles (EVs), subsequently activating the STING pathway in the recipient cells and potentiating antitumor immune responses [69]. However, this concept remains debated in cellular senescence. During aging, the permeability of the cell membrane is markedly perturbed, which frequently coincides with the onset of apoptosis, promoting efflux of cellular constituents. Consequently, it is plausible that cGAMP is extruded into the senescence-associated microenvironment via the first mechanism. Recent investigations have uncovered that apoptotic T cells release EVs bearing surface ENPP1 that hydrolyzes extracellular cGAMP in radiation enteritis, which attenuates STING-mediated immune responses and alleviates tissue damage [70]. However, the molecular mechanisms governing cGAMP sorting into EVs and the functional consequences of EV-associated versus free cGAMP require further exploration. In the context of cellular senescence, EVs are now recognized as a novel component of the SASP [71], facilitating paracrine signalling that can induce senescence in adjacent cells. EVs can encapsulate various cargoes, including microRNAs and proteins, acting as systemic messengers [72–74]. Therefore, EVs represent a plausible and significant mechanism for cGAMP-mediated intercellular communication in the senescence-associated microenvironment. Similarly, amphisomes, formed by the fusion of autophagosomes with multivesicular bodies, have been implicated in the release of mitochondrial DNA (mtDNA) during the process of inflammatory aging, raising the intriguing possibility that cGAMP might also utilize this pathway. In older individuals, activated T cells can discharge significant quantities of mtDNA, linked to the fusion of these hybrid vesicles with the plasma membrane and the subsequent extracellular release of their contents [75]. While this study focuses on the CISH-lysosome-mtDNA-inflammaging axis, the vesicular release mechanism suggests a potential parallel pathway for cGAMP extrusion. These findings elicited an intriguing possibility that cGAMP may serve as a mediator in immune communication in the aging microenvironment through the conveyance of exosomes from source cells to adjacent cells. This proposed mechanism offers a novel perspective for investigating the extracellular transport dynamics of cGAMP, thus enriching the discourse on cellular senescence and intercellular signalling (Fig. 2). Together, these diverse export mechanisms—ranging from active transport to passive release and vesicular packaging—underscore the versatility of cGAMP as an intercellular messenger. However, for intercellular signalling to be completed, extracellular cGAMP must be efficiently recognized and internalized by recipient cells, a process mediated by an equally sophisticated array of import mechanisms that we examine next.

### Uptake and sensing pathways of cGAMP

For intercellular signalling to be completed, extracellular cGAMP must be efficiently internalized by recipient cells, a process mediated by a sophisticated array of import mechanisms. The propagation of intercellular immune signalling depends on efficient recognition and internalization of extracellular cGAMP by recipient cells. Multiple complementary uptake mechanisms operate with distinct cell-type specificities and regulatory properties (Fig. 2). The solute carrier (SLC) superfamily provides the principal route for cGAMP internalization: SLC19A1 (Solute Carrier Family 19 Member 1), formerly known as the reduced folate transporter, functions as a Na⁺-coupled folate/riboflavin importer that mediates cGAMP uptake in diverse cell types [67, 76]. Its broad tissue distribution enables widespread cellular responsiveness to extracellular cGAMP signals. Notably, SLC19A1 expression is upregulated by Type I interferons, establishing a positive feedback loop that amplifies cGAMP-mediated immune responses. Structurally, SLC19A1 is a 12-transmembrane helix protein [77]. At high extracellular cGAMP concentrations, an outward-open conformation is adopted for its 12-transmembrane helix bundle. Residues Arg252, Tyr236, and Gln138 coordinate to form a high-affinity binding pocket where cGAMP binds in a head-to-tail dimeric arrangement [77]. Subsequent proton binding triggers a conformational transition from a closed to an open state, facilitating cGAMP release—a process energized by the proton gradient. In contrast, SLC46A2 exhibits a restricted expression pattern. It is selectively highly expressed by cells of the mononuclear-macrophage lineage, including macrophages and dendritic cells [78]. This specialization suggests distinct roles in coordinating innate immune cell activation during cGAMP-mediated immune surveillance. The proton-driven transport mechanism of SLC46A2 may enable efficient cGAMP uptake in acidic microenvironments characteristic of inflammatory sites and tumors. The differential expression of SLC19A1 and SLC46A2 across cell types creates a hierarchical responsiveness to extracellular cGAMP. Professional antigen-presenting cells exhibit enhanced uptake capacity. Despite belonging to the same SLC family, SLC46A2 employs a distinct transport mechanism involving key residues Arg155, Tyr185, and His232 [78]. These structural differences result in a binding pocket with a smaller capacity and consequently a lower maximum flux compared to SLC19A1. The differential expression and transport properties of these two transporters create a hierarchical responsiveness to extracellular cGAMP. Professional antigen-presenting cells exhibit enhanced uptake capacity through SLC46A2-mediated transport. Alternatively, cGAMP can permeate the membrane through non-specific conduits that operate with distinct activation mechanisms and physiological contexts. The ATP-gated P2X7 purinergic receptor (P2X7R) functions as a ligand-gated cation channel. In tumor microenvironments, P2X7R on macrophages is shown to mediate cGAMP import when extracellular ATP levels are elevated, amplifying interferon responses in a MerTK-regulated manner [79]. P2X7R has been shown to operate via a dual-gating mechanism: ATP binding initially opens the canonical ion channel, and sustained stimulation induces C-terminal/lipid raft rearrangements, leading to pore dilation [80, 81]. This large pore formation enables passive diffusion of cGAMP across the plasma membrane. Notably, P2X7R activation is associated with inflammasome signalling, suggesting potential crosstalk between cGAMP and IL-1β pathways. P2X7R thus provides a damage-coupled cGAMP import pathway: During conditions of elevated extracellular ATP—such as infection, chemotherapy, or necrotic cell death—channel opening facilitates cGAMP uptake and amplifies interferon responses in bystander cells [60, 82]. The Leucine-Rich Repeat-Containing Protein 8 (LRRC8) volume-regulated anion channels (VRAC) mediate cGAMP permeation in response to osmotic stress [83]. LRRC8-mediated cGAMP transport has been implicated in antiviral defense and cancer immunotherapy responses. The LRRC8 channel comprises a hexameric assembly of the essential LRRC8A subunit with accessory subunits (LRRC8C/E or LRRC8D). Channels containing A/C/E subunits support cGAMP permeation, whereas A/D-type channels exhibit a constricted pore diameter that hinders cGAMP passage [83, 84]. Upon hypotonic stimulation, increased membrane tension induces hexamer expansion, resulting in a transient inward current [84]. Net cGAMP import occurs when its extracellular concentration exceeds the intracellular levels, enabling VRAC to facilitate bidirectional, concentration gradient-driven signal transmission between tumor and immune cells. Distinct from the ATP-dependent gating of P2X7R, LRRC8-mediated cGAMP transport has been implicated in antiviral defense and cancer immunotherapy responses. Furthermore, the antimicrobial peptide LL-37 promotes internalization by forming membrane-active complexes with cGAMP [61]. LL-37 is not a traditional active transporter but rather an amphipathic α-helical host defense peptide. It facilitates the uptake of extracellular cGAMP, potentially via complex formation and endocytosis [61]. Specifically, the positively charged LL-37 and the tetranegative cGAMP form a 1:1 electrostatic complex. This complex may recruit adjacent LL-37 and cGAMP molecules, leading to the formation of peptide-nucleotide condensate particles [61]. These particles appear to enter cells through lipid raft-dependent mechanisms, although the precise route of cytosolic delivery remains unclear. Notably, this LL-37-cGAMP complex is susceptible to hydrolysis by ENPP1, providing a regulatory checkpoint in extracellular cGAMP signalling [60]. In addition to these transmembrane routes, cGAMP is internalized through vesicular pathways, including fluid-phase endocytosis and receptor-mediated uptake of cGAMP-containing exosomes, particularly in professional antigen-presenting cells such as dendritic cells and macrophages (Fig. 2). For instance, exosomes encapsulating cGAMP engage integrin CD11c and C-type lectin receptors CD205 on the surface of dendritic cells [43]. This interaction triggers clathrin and AP2 assembly, leading to membrane invagination and clathrin-coated pit formation. GTP hydrolysis by dynamin drives vesicle scission, generating early endosomes (EEA1-positive) [43]. Progressive endosomal acidification by V-ATPase induces membrane destabilization, resulting in cGAMP release into the cytosol for STING activation [43]. Additionally, macrophage phagocytosis of cGAMP-containing tumor debris constitutes another import mechanism for nucleic acid sensing [85, 86]. When cGAMP-bearing exosomes or cGAMP-protein complexes released by apoptotic/necrotic tumor cells are opsonized by complement or IgG, FcγR (CD32/64) or MerTK on macrophages recognize these targets [43]. This recognition subsequently activates the RhoA-ROCK and Rac1-WAVE pathways, inducing pseudopod extension to engulf the target and internalize cGAMP [43]. Building on these insights, engineered nanocarriers have been developed to mimic exosomal delivery, achieving enhanced intracellular retention of cGAMP [87].

In summary, the cGAMP transport network comprises major import pathways—SLC19A1, SLC46A2, P2X7R, and LL-37—and dual export pumps—ABCC1—complemented by gap junctions, EVs, and the ENPP1 degradation machinery. These pathways demonstrate high specialization in molecular recognition, energy coupling, and gating kinetics, but they are interconnected through cross-regulatory signals such as concentration gradients, membrane potential, and extracellular ATP. This multicomponent, druggable transport system not only elucidates the mechanistic basis for cGAMP’s intercellular trafficking, but also provides precise targets for enhancing CDN-based immunotherapy and reversing tumor immune evasion. Over the next decade, with the refinement of structure–function maps and the integration of multi-omics data, the mechanism of cGAMP transport is projected to become a classic paradigm at the intersection of immunometabolism and membrane biology.

## Physiological roles of extracellular cGAMP

Having established the molecular and cellular mechanisms that enable cGAMP to traverse tissue boundaries, we now turn to the functional consequences of this intercellular communication. The elaborate transport systems described above enable cGAMP to exert diverse physiological effects beyond its cell of origin, functioning not merely as a danger signal but as a sophisticated microenvironmental modulator. Under steady-state conditions, the intracellular synthesis of cGAMP is minimal, resulting in extremely low circulating levels. Elevated cGAMP in blood or tissues typically indicates pathological states, including inflammatory disorders, infections, and malignancies [88–91]. For effective STING activation, a threshold concentration of cGAMP is required, though the precise level remains poorly defined and likely varies across pathological contexts. Upon intercellular transfer, cGAMP propagates along spatiotemporal gradients, undergoes stepwise amplification, and is precisely coordinated—ultimately becoming integrated into long-range regulatory networks that govern tissue repair and homeostasis. The concentration and duration of cGAMP exposure and the specific identity of responding cells collectively determine the functional outcome of the immune response, ranging from protective immunity to immunopathology. This section examines how cGAMP orchestrates antiviral defenses, maintains tissue homeostasis, and participates in the resolution of sterile inflammation.

### Orchestration of antiviral immunity

Recent studies establish extracellular 2′3’-cGAMP as a mobile, second-messenger alarm that exerts overwhelmingly beneficial effects in antiviral immunity [30, 92]. cGAMP not only activates the cGAS-STING axis in myeloid cells to release inflammatory cytokines that recruit and activate NK and T cells, thus potentiating viral clearance, but also spreads via multiple routes—protein transporters, gap junctions, or virion/vesicle encapsulation—amplifying Type-I interferon signalling and synchronizing a multicellular antiviral shield long before viral replication peaks [93–95]. For example, progeny virions produced in cGAS-positive cells can package 2′3’-cGAMP and deliver it to naïve neighbouring cells; and these cells then trigger STING-IRF3-dependent IFN production without ever sensing DNA, thus pre-arming the tissue [96, 97]. This preemptive intercellular alert system represents a sophisticated evolutionary adaptation for host defense, illustrating how cGAMP’s transport capabilities transform localized DNA sensing into tissue-wide immune protection.

### Maintenance of tissue homeostasis and repair

Beyond acute infection control, cGAMP also plays nuanced roles in tissue maintenance and repair. Rather than functioning as a simple on/off switch for inflammation, cGAMP operates as a “rheostat” for tissue homeostasis: The same pathway can toggle between pro- and anti-inflammatory states, or between quiescent and stem-cell-activating states, depending on its dose, duration, and the target cell type. In periodontitis, mitochondrial DNA leakage during inflammation activates cGAS, leading to excessive cGAMP accumulation in periodontal ligament stem cells (PDLSCs); and chronic STING activation consequently arrests osteogenic differentiation. A multifunctional injectable hydrogel system that simultaneously inhibits cGAMP synthesis, facilitates its export, and promotes enzymatic degradation restores cGAMP homeostasis and enables full regeneration of alveolar bone in 4 weeks [98]. In metabolic disease, cGAMP acts as an “immuno-metabolic tuner” [99, 100]: It non-canonically suppresses multi-tissue inflammation by inhibiting LPS-induced JNK/p65 phosphorylation in macrophages, while enhancing insulin signalling via an Akt-dependent pathway, thus reducing hepatic glucose output and lipid deposition and ameliorating high-fat-diet-induced metabolic dysfunction. Moreover, some researchers reveal a U-shaped relationship between cGAMP levels and infarct size—both deficiency and excess increase myocardial infarction risk—indicating that cGAMP serves as a damage-resolution signal that restrains excessive inflammation [101]. Similarly, in hepatic ischaemia–reperfusion injury, elevated cGAMP activates STING, leading to down-regulation of global protein translation through the non-canonical PERK-eIF2α axis, suppression of ROS production, and marked attenuation of cellular senescence and subsequent fibrosis [100, 102]. These findings illustrate the context-dependent nature of cGAMP signalling, where its ultimate effect is determined by the specific tissue environment and the nature of the insult—a theme that becomes particularly relevant when considering its roles in chronic inflammatory and age-related conditions.

### Implications in sterile Inflammation

The dual nature of cGAMP signalling is particularly evident in the context of sterile inflammation. On one hand, excessive or sustained cGAMP production serves as a key driver of chronic inflammation. On the other hand, transient, low-level cGAMP signalling can restrain excessive inflammatory amplification through negative feedback mechanisms, thus exerting anti-inflammatory effects. Specifically, controlled activation of the cGAMP-STING pathway triggers a moderate inflammatory response that enhances local immune surveillance and facilitates clearance of senescent or dysfunctional cells. For instance, physiological or low-concentration cGAMP can upregulate IL-10 via the STING-IRF3 axis [103, 104]. Moreover, the STING-ULK1 pathway activates mitophagy to clear leaked mtDNA, limiting excessive cGAMP production and establishing a negative feedback loop [104, 105].

In summary, the anti-inflammatory effect of cGAMP does not reflect merely the absence of pro-inflammatory signalling; rather, it represents an active homeostatic process mediated through the STING negative feedback circuit under conditions of transient, low-level stimulation, thus restoring inflammatory homeostasis. However, once cGAMP concentration or signalling duration exceeds a physiological threshold, the same molecule switches to function as a pro-inflammatory driver—a transition whose molecular mechanisms will be elaborated in subsequent sections. This delicate balance renders cGAMP both an initiator and a regulator of inflammatory processes. Understanding these physiological roles provides essential context for evaluating its pathological dysregulation, to which we now turn.

## Roles of cGAMP in pathological diseases

The physiological functions of cGAMP as an immune modulator and tissue homeostasis regulator provide a framework for understanding its involvement in disease pathogenesis. When the finely tuned balance of cGAMP signalling is disrupted—whether through excessive production, impaired degradation, or aberrant transport—the consequences can be profound and varied. We focus on four major disease categories—infectious diseases, cancer, autoimmune disorders, and aging-related conditions—not merely because of their clinical prevalence, but because they represent distinct pathological contexts in which cGAMP signalling exhibits characteristic patterns of dysregulation. In infectious diseases, cGAMP functions mainly as a beneficial defense signal; in cancer, its effects are paradoxically context-dependent, promoting both antitumor immunity and tumor progression; in autoimmune diseases, self-DNA-driven cGAMP production breaks immune tolerance; and in aging, chronic cGAMP signalling drives sterile inflammation and cellular senescence. These four categories thus span the spectrum from acute to chronic conditions, from protective to pathological outcomes, and from extrinsic to intrinsic triggers—enabling a comprehensive analysis of cGAMP’s disease relevance.

### Infectious diseases

**Fig. 3:**
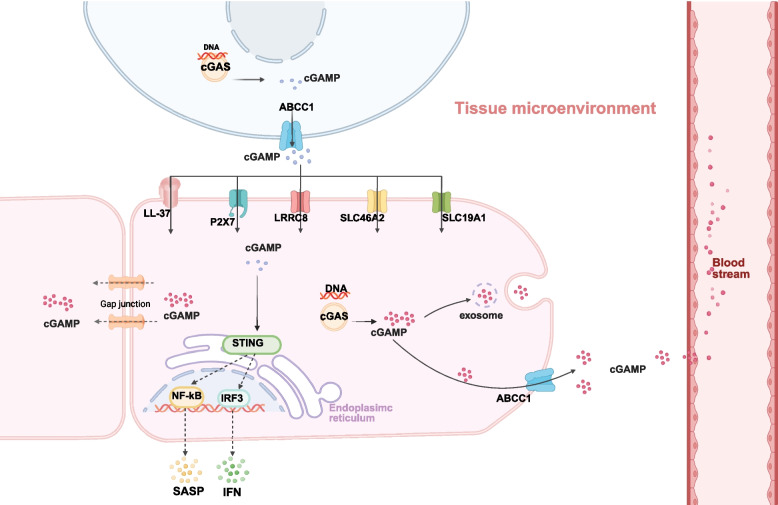
cGAMP in different microenvironments. cGAMP has dual functions in different tissue microenvironments, mainly manifested as antiviral, anti-tumor or pro-tumor, participating in maintaining tissue homeostasis and repair, and mainly playing a pro-inflammatory role in the aging microenvironment

The cGAMP-STING-IFN axis operates effectively against both DNA viruses and RNA viruses that generate DNA intermediates [106–108], providing a mechanistic basis for developing cGAMP-based broad-spectrum antiviral strategies. In antiviral research, co-culture experiments by Pepin et al. demonstrated that epithelial cells directly transfer cGAMP to macrophages via gap junctions, thus activating the STING signalling pathway in recipient cells [97]. During HIV-1 infection, cGAMP promotes intercellular transfer at the membrane fusion sites between T lymphocytes and macrophages, amplifying STING signalling in macrophages [109]. Beyond direct antiviral effects, cGAMP exerts immunomodulatory and hypotensive functions during infection. For instance, co-culture experiments with colonic macrophages and intestinal epithelial cells (IECs) revealed a synergistic positive-feedback loop, wherein cGAMP enhances interleukin (IL)−18 secretion by IECs, which in turn stimulates colonic macrophages to produce substantial amounts of cGAMP [110]. During endotoxemia, endothelial cells release cGAMP via volume-regulated anion channels (LRRC8C), which acts on adjacent vascular smooth muscle cells to activate the STING-PKG-I pathway, facilitating vasorelaxation [111] (Fig. 3). These diverse actions during infection highlight the broad therapeutic potential of modulating cGAMP pathways, while raising important considerations about how chronic activation might contribute to inflammatory sequelae—a concern that becomes particularly acute in the context of cancer, where cGAMP exhibits a striking functional duality.

### Cancer

In cancer, the role of cGAMP is particularly complex, exhibiting both antitumor and pro-tumor activities depending on the context. In the tumor microenvironment, genomic instability and replication stress induce DNA damage, leading to spontaneous cGAS activation and continuous cGAMP production [43, 112, 113]. Moreover, the antitumor effects induced by radiotherapy or anthracycline-based chemotherapy also depend on their ability to trigger the release of cGAMP from tumor cells [86, 114]. Once it is released into the extracellular space, cGAMP can be taken up by antigen-presenting cells (such as dendritic cells and DCs) or immune cells (such as macrophages) in the tumor via gap junctions, exosomes or other intercellular routes. Inside DCs, cGAMP activates the STING pathway, which not only promotes DC maturation and Type I interferon secretion but also significantly improves their ability to cross-present tumor antigens by upregulating the costimulatory molecule and the chemokine [115–118]. This process efficiently activates tumor-specific CD8⁺ T cells. For instance, in both in vitro and in vivo tumor experiments, cGAMP-induced IFN signalling transcriptionally upregulates TCF1, maintaining the stem-like properties and secondary expansion potential of CD8⁺ T cells [43]. Additionally, cGAMP also activates the STING-NLRP3 pathway in macrophages, leading to IL-18 and IL-1β production, which subsequently induces 4-1BBL/4-1BB co-stimulatory signalling to optimize NK cell antitumor function [119]. Furthermore, cGAMP released by tumor cells can activate STING signalling in endothelial cells, promoting transendothelial migration of lymphocytes [120]. Thus, extracellular cGAMP serves as a critical bridge connecting cancer cells with both the innate and adaptive immune systems, playing an essential role in initiating and coordinating effective antitumor immune responses.

However, persistent intracellular cGAMP in tumor cells can also promote tumor progression and immune suppression. For instance, studies have shown that chronic cGAMP-STING signalling induces non-canonical NF-κB activation, facilitating tumor cell survival, metastasis, and drug resistance [113, 117, 121]. Moreover, in many tumor models, long-term activation of this mechanism has been found to foster an immunosuppressive microenvironment [122, 123]. For example, excessive cGAMP uptake by T cells can trigger STING-mediated cell death or functional exhaustion, compromising antitumor immunity [124] (Fig. 3). This context-dependent duality poses significant challenges for therapeutic targeting while offering opportunities for precision intervention in cancer. The parallels between cancer and autoimmune diseases are instructive: In both conditions, cGAMP signalling becomes dysregulated in response to self-derived nucleic acids, though the immunological consequences differ markedly depending on whether the response is directed against transformed cells or healthy tissues.

### Autoimmune and autoinflammatory diseases

Autoimmune disorders often arise from a breakdown in immune tolerance and heightened immune responses [125]. These conditions are characterized by the immune system misidentification of the body’s own tissues as “foreign,” resulting in chronic inflammation, tissue damage, and even organ failure [126, 127].

cGAMP does not initiate autoimmune pathology independently; rather, as the central second messenger of the cGAS-STING pathway, it functions as a potent amplifier when self-DNA is misrecognized as pathogenic, driving or exacerbating multiple autoimmune conditions through excessive Type I interferon production. Specifically, triggered by infection, mitochondrial damage, or genetic mutations such as TREX1 deficiency, self-DNA becomes aberrantly exposed [91, 128, 129]. This initiates a self-amplifying cycle with two reinforcing arms: On one hand, activation of autoreactive B and T cells leads to autoantibody production and inflammatory cytokine release; on the other hand, cGAS recognition of exposed DNA generates excessive cGAMP, which further amplifies inflammation via STING, thus exacerbating autoimmune pathology. In certain autoimmune diseases, including rheumatoid arthritis (RA) and systemic lupus erythematosus (SLE), self-DNA is aberrantly released, particularly from apoptotic cells [130, 131]. This triggers cGAMP production and STING activation, leading to excessive Type I IFN production [132, 133]. Elevated IFN-I levels subsequently amplify immune responses, causing immune-mediated damage to healthy tissues [134]. Using mass spectrometry, elevated cGAMP levels have been detected in the peripheral blood of SLE patients [135]. Thus, cGAMP serves as a critical amplifier in the self-sustaining cycle of autoimmune pathogenesis. The chronic, systemic nature of autoimmune inflammation shares important features with the sterile inflammation characteristic of aging, suggesting common mechanistic underpinnings that we explore in the following section.

### Aging and age-related diseases

The role of cGAMP in aging represents one of the most compelling and rapidly evolving areas of research. Senescent cell accumulation is a major contributor to organismal aging. The immune system, particularly the innate immune response, functions as a natural defense mechanism against senescent cells [136]. By augmenting the functionality and surveillance capacity of immune cells, it bolsters their phagocytic clearance of senescent cells [137].

Recent studies have unveiled a novel biological function of cGAMP, which is its capacity to regulate cellular migration through the STING-independent RAB18-FOSB signalling pathway. This non-canonical pathway operates independently of STING activation, offering new perspectives for its potential applications in the study of aging or in clinical settings [138]. In contrast, the canonical STING-dependent functions of cGAMP are notably involved in the development of multiple conditions, including progeria syndrome, age-related cardiovascular inflammation [139–142], and neuroinflammation [105]. In these contexts, cGAMP triggers the STING activation, promoting the release of pro-inflammatory mediators, such as tumor necrosis factor (TNF)-α and IL-6 from macrophages and endothelial cells. These mediators intensify local inflammation in the vascular wall, contributing to the atherosclerotic plaque formation [143, 144] and activation of microglia and astrocytes [105].

Furthermore, chronically elevated cGAMP was detected in multiple organs of aged mice, where persistent STING phosphorylation drives the SASP. Genetic deletion of cGAS or pharmacological inhibition of STING with H151 reverses peripheral organ atrophy and improves neuro-cognitive performance, implying that age-related functional decline can be delayed by lowering cGAMP [145]. In neurodegenerative diseases, neurons often exist under conditions of chronic cellular stress, characterized by oxidative stress, mitochondrial dysfunction, and accumulation of misfolded proteins [146–148]. These stressors can cause DNA damage in neurons, which in turn stimulates cGAMP production and triggers inflammatory responses [105, 149]. The accumulation of misfolded proteins, such as β-amyloid (Aβ) in Alzheimer’s disease and α-synuclein in Parkinson’s disease, further exacerbates this situation [148]. Moreover, the transfer of cGAMP from neurons to microglia, leading to the activation of Type I IFN responses, constitutes a pivotal molecular mechanism that amplifies neuroinflammation and promotes neuronal apoptosis [150]. Notably, cGAMP can also upregulate TREM2, facilitating microglial transition from a pro-inflammatory phenotype to an anti-inflammatory phenotype, thus alleviating neuroinflammation associated with sleep deprivation [151]. This dual role highlights the context-dependent nature of cGAMP signalling in the aging brain, echoing the functional complexity observed in cancer and autoimmune diseases.

Previous research has indicated that the fusion efficiency between bone marrow-derived cells (BMDCs) and somatic cells is significantly enhanced under conditions of chronic inflammation, aging, and tissue injury [152, 153]. This implies that membrane fusion between cells may occur under specific conditions, such as aging (Fig. 3). Therefore, cGAMP appears to play an intricate role in the aging microenvironment. To systematically address this complexity, we have thoroughly examined several potential mechanisms by which cGAMP influences cellular senescence and summarize them as follows:

#### Surveillance of intracellular DNA damage

**Fig. 4:**
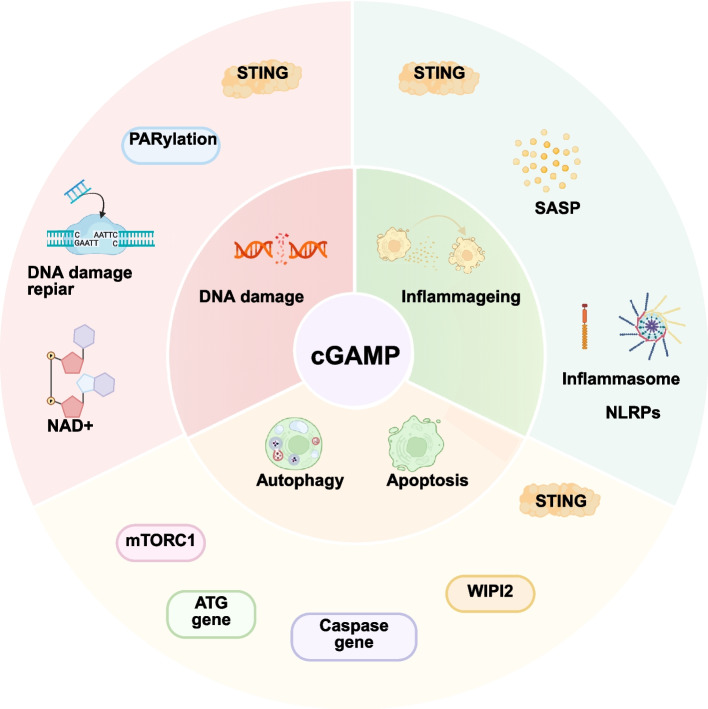
cGAMP in aging microenvironments. The mechanisms underlying the role of cGAMP in cellular senescence and damage primarily encompass DNA damage, senescence-associated inflammation, autophagy, and apoptosis. Specifically, the DNA damage mechanism involves the activation of the DNA damage response (DDR), inhibition of PARylation, and depletion of NAD⁺ levels. Senescence-associated inflammation is mainly characterized by the involvement of the senescence-associated secretory phenotype (SASP) and inflammasomes. Autophagy and apoptosis interact with each other and collectively participate in the regulation of cellular senescence

As cells age, their DNA may accumulate damage during replication and repair processes, particularly due to telomere erosion, oxidative stress, and other endogenous stressors [154]. These endogenous stress factors can lead to DNA leakage from the nucleus and mitochondria into the cytoplasm [155]. During aging, compromised nuclear-envelope integrity permits nuclear DNA to leak into the cytoplasm as cytoplasmic chromatin fragments (CCFs). These fragments have been identified as potent drivers of the cGAS-STING-SASP axis, and age-dependent down-regulation of their cognate nucleases further amplifies CCF accumulation, establishing a self-perpetuating vicious cycle [19, 156]. Cytoplasmic DNA promotes cGAMP synthesis and subsequently activates DNA-damage-associated inflammation and cellular senescence [157]. Downstream of cGAMP signalling, IFN-I promotes cellular senescence by amplifying the DNA damage response (DDR) and activating the p53 pathway [158]. Additionally, cGAMP can activate DDR signalling in a manner independent of the canonical IFN pathway by inhibiting polyADP-ribosylation (PARylation) [159], which is a post-translational modification catalyzed by enzymes in the PARP family that facilitate the recruitment of DNA-repair factors [160]. Concurrently, cGAMP can lower the intracellular NAD^+^ levels [159], which serves as the essential ADP-ribose donor for PARylation reactions [161], and NAD^+^ depletion has been implicated in aging, age-related inflammation, and neurodegenerative conditions [162, 163]. Thus, cGAMP not only serves as a sentinel biomarker of cellular DNA damage, but also signifies the entry of senescent cells into an active immune state (Fig. 4).

#### Aging-related inflammation

Aging-related inflammation is characterized by a gradual decline in cellular functionality and the development of a chronic, low-grade inflammatory state that occurs as part of the aging process. Unlike typical inflammatory responses that arise from infections or injuries, chronic low-grade inflammation is driven by a complex interaction of endogenous factors, and the mechanisms involved are likely linked to the SASP, immunosenescence, and oxidative stress [164–166]. Cells undergoing senescence often release a range of substances collectively known as the SASP, such as cytokines, chemokines, growth factors, and proteases [167, 168]. An increasing body of research is targeting the cGAS-cGAMP-STING signalling pathway as a potential intervention focusing on inflammaging [169]. STING-mediated activation of the NF-κB pathway by cGAMP can result in the secretion of numerous SASP factors, including IL-1β, matrix metalloproteinases, and granulocyte–macrophage colony-stimulating factor [170, 171]. cGAMP also plays a role in inflammasome activation, particularly involving the NOD-like receptor family pyrin domain-containing proteins (NLRPs), such as NLRP1 and NLRP3 [172, 173]. NLRP1 inflammasomes are integral to the senescence process, mediating SASP release in a manner dependent on gasdermin D, a protein that is crucial for regulating cell death [173]. Moreover, NLRP3 activation can stimulate the secretion of IL-1β [174], which promotes chronic inflammatory responses in the organism. As individuals age, the decline in immune functionality and surveillance capability hampers the body’s ability to eliminate senescent cells effectively. The cGAMP-STING pathway may enable senescent cells to evade immune detection in part, thus prolonging inflammatory responses and contributing to chronic, low-grade inflammation. Furthermore, excessive cGAMP activation may trigger sustained inflammatory reactions, predisposing to age-related pathologies. In summary, cGAMP elicits senescence-associated inflammatory responses by facilitating SASP secretion, activating the inflammasome complex, and inducing immunological senescence (Fig. 4). Through these mechanisms, cGAMP serves as a key molecular link between cellular senescence and the systemic inflammatory state of aging.

#### Autophagy and apoptosis

cGAMP facilitates apoptosis and autophagy, either directly or indirectly. cGAMP stimulates effector T cells to produce higher levels of IFN-I than those produced by innate immune cells. The induction process is contingent upon activation of the mechanistic target of rapamycin complex 1 (mTORC1). mTORC1 serves as a central regulatory molecule in cell growth and an inhibitor of autophagy [175, 176], playing a pivotal role in the regulation of cellular proliferation and autophagy processes. cGAMP can partially counteract the activity of mTORC1, thus inhibiting cell growth and proliferation [177]. Additionally, Gui et al. have elucidated that cGAMP-induced cellular autophagy operates independently of the canonical autophagy pathway (i.e., TBK1-IRF3). Instead, it facilitates the lipidation of LC3, a marker of autophagy, through a pathway that is contingent upon WIPI2 and ATG5, thus playing a pivotal role in antiviral responses [178].

cGAMP not only modulates cellular autophagy but also participates directly or indirectly in regulation of apoptosis. During STING activation, cGAMP indirectly modulates the expression of autophagy-related genes, such as ATG family members [179], thus enhancing the autophagic flux. Additionally, cGAMP can activate transcription factors, including IRF3 and NF-kappa B, via TBK1-mediated phosphorylation, leading to the expression of apoptosis-related genes and subsequent activation of caspases [180], and thus promoting cellular apoptosis. Notably, exogenous cGAMP can directly activate STING, inducing mitotic arrest and cell death [181]. A complex interplay exists between autophagy and apoptosis [182, 183] (Fig. 4). In certain contexts, autophagy may inhibit apoptosis, while dysregulated autophagy may exacerbate cell death in others. By enhancing autophagy, cGAMP may also evoke apoptotic responses by modulating STING signalling pathways. Consequently, the role of cGAMP in the determination of cellular fate is multifaceted and context-dependent. Nonetheless, the originating cells and the precise roles of cGAMP in senescence remain poorly defined, requiring further investigation.

## Therapeutic targeting of cGAMP

**Fig. 5:**
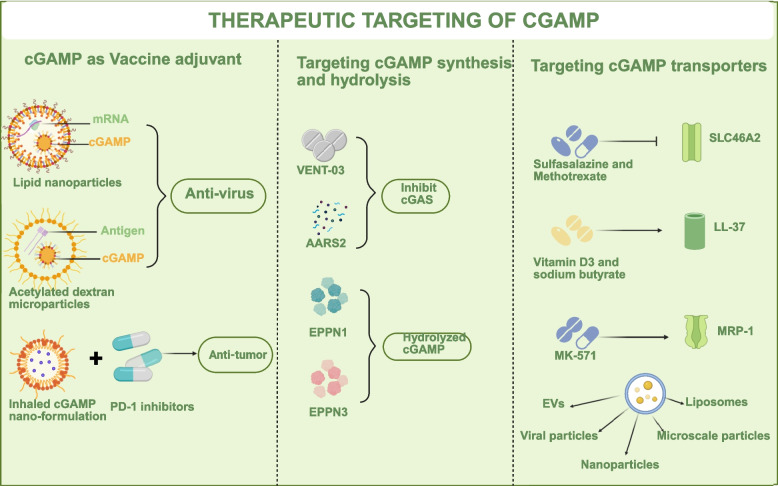
cGAMP targeting strategy. cGAMP itself can serve as an adjuvant for therapeutic drugs and be delivered by encapsulation through liposomes, nanoparticles or membrane vesicles, which can further enhance antiviral and anti-tumor efficacy. Targeting the synthesis and degradation of cGAMP is a therapeutic strategy. The development of small molecules acting on cGAMP transporters can enhance or weaken the signaling effect of cGAMP for different diseases

The diverse pathological roles of cGAMP across infectious diseases, cancer, autoimmunity, and aging establish it as a compelling therapeutic target. Mechanistic investigations must ultimately facilitate therapeutic intervention. As cGAMP constitutes the central node of the cGAS-STING pathway, it has emerged as a promising target with multimodal intervention possibilities. Moreover, we propose that targeting cGAMP (rather than cGAS or STING) should be regarded as a more advantageous therapeutic strategy for disease treatment. As the direct regulator in the pathway, exogenous administration or degradation of cGAMP enables transient activation or suppression of the entire signalling cascade. In contrast, to inhibit cGAS, prior blockade of DNA recognition is required, while STING inhibition depends on the turnover of pre-existing protein pools—both exhibiting significantly slower kinetics. Furthermore, to achieve comparable levels of STING phosphorylation, a lower dose threshold for cGAMP is required compared to agents targeting cGAS or STING. Consequently, pharmacological interventions directed at cGAS or STING typically demand higher drug exposure, thus elevating the risk of adverse effects and toxicity. Thus, targeting the cGAMP axis (ranging from synthetic analogues, pro-drug nanocarriers, and antibody–drug conjugates to CRISPR-based gene circuits) offers a broad immunotherapeutic platform for multiple diseases (Fig. 5).

### cGAMP as a vaccine adjuvant

Independent studies have established that 2’,3’-cGAMP functions as a potent self-adjuvanting agent capable of amplifying both humoral and T cell responses [184]. Formulations with cGAMP either co-encapsulated with mRNA encoding viral antigens in lipid nanoparticles (LNPs) [185–188] or embedded in acetylated dextran (Ace-DEX) microparticles together with a universal COBRA HA antigen have shown superior efficacy against influenza challenge [189]. The evolutionary conservation of cGAMP as a “danger” signal—from the bacterial CBASS anti-phage system to mammalian interferon responses—further supports its utility as a broad-spectrum antiviral molecular adjuvant [190]. Notably, cGAMP nano-formulation has already advanced to early-phase clinical testing, where it reverses the “cold” tumor micro-environment and significantly delays tumor growth [123, 191]. Moreover, clinical studies have demonstrated that combined administration of cGAMP restores the efficacy of PD-1 inhibitors in cGAS-deficient tumor models [112]. Additionally, investigations have revealed that cGAMP plus 5-FU not only potentiates tumor suppression but also attenuates systemic 5-FU toxicity, indicating its potential as an immune-chemotherapeutic strategy for sensitization with reduced adverse effects [192] (Fig. 5). These applications demonstrate the practical utility of cGAMP in enhancing immune activation in vaccination and cancer therapy.

### Targeting cGAMP synthesis and hydrolysis

Alternatively, given the crucial role of cGAMP in immune modulation, therapeutic strategies can focus on modulating endogenous cGAMP levels by targeting its synthesis or degradation. cGAS is identified as the principal enzyme responsible for cGAMP production. Consequently, regulation of cGAS activity serves as a primary strategy for controlling cGAMP levels. Numerous small-molecule inhibitors and gene-editing technologies have been developed to mitigate diseases driven by hyperactive immune responses, including inflammation related to aging and autoimmune disorders, by inhibiting the catalytic activity of cGAS, and thereby reducing cGAMP synthesis, ultimately achieving anti-inflammatory effects. For instance, VENT-03 (NCT07260877) has been identified as the first cGAS inhibitor to progress into Phase II clinical trials [193–196] (Table 1). It has reported that VENT-03 has demonstrated good safety and tolerability at all tested dose levels. Alanyl-tRNA synthetase (AARS2) inactivates cGAS through lactylation, thus inhibiting cGAMP synthesis and innate immune activation [197] (Table 1).

**Table 1:** Regulation and Intervention Strategies of cGAMP

Target	Intervention Modality	Medicine	Development Stage and NCT number	Specific Molecular Mechanism	Effect	Therapy
cGAS	cGAS inhibitors	VENT-03	Phase II clinical trial (NCT06747821) [193–196]	Allosteric inhibition of catalytic domain of cGAS; ATP/GTP substrate blockade	Inhibit cGAMP synthesis and STING signalling	Anti-inflammatory
		AARS2 [197]	Preclinical research	Lactylation-dependent cGAS inactivation		
cGAMP Hydrolase	ENPP1 inhibitors	SR-8314 or MV-626 [198–201]	Preclinical research	Competitive inhibition of catalytic site of ENPP1	Inhibit cGAMP hydrolysis and enhance STING signalling	Antitumor
		RBS2418	Phase Ia/b clinical trial (NCT05270213) [202]; Phase II clinical trial (NCT06824064) [203]			
		SR-8541A	Phase I clinical trial (NCT06063681) [204]; Phase II clinical trial (NCT06589440) [204]			
		TXN10128	Phase I clinical trial (NCT05978492) [205, 206]			
		ISM5939	Phase I clinical trial (NCT06724042) [207, 208]			
		Methotrexate (MTX) [209]	Preclinical research	MTX competitively binds to the substrate-binding pocket of ENPP1		
	SLC46A2	Sulfasalazine (SSZ) [67, 76, 78]	Repurposing	Direct inhibition of SLC46A2-mediated cGAMP import		
	LL-37 inducer	Vitamin (VD3) [61, 210]	Preclinical research	Transcriptional upregulation of LL-37 via VDRE	Enhance cGAMP transport and STING signalling	Immunomodulation; alleviate inflammation
		Sodium butyrate [61, 211]	Preclinical research	Epigenetic activation of the LL-37 promoter		
	Inhibit MRP1(ABCC)	MK-571 [15, 64]	Preclinical research	Competitive inhibition of ABCC1 nucleotide-binding domain	Inhibit cGAMP efflux to activate STING signalling	Anti-inflammatory
	Encapsulate cGAMP for targeted delivery	EVs [212–214]	Preclinical research	Mimicking natural intercellular transfer	Deliver cGAMP to activate STING signalling	Immunomodulation; antitumor
		Viral particles [215]	Preclinical research	cGAMP-loaded as a vaccine adjuvant		
		Microscale [189, 214, 216–218]	Preclinical research	Sustained release of cGAMP for persistent local STING activation		
		Lipid nanoparticles [219–221]	Preclinical research	Encapsulation protects cGAMP from degradation by ENPP1		

By targeting the synthase, hydrolase, and transporter of cGAMP, the intracellular and extracellular levels of cGAMP can be modulated, thereby exerting corresponding immunomodulatory effects and anti-inflammatory functions

Degradation of cGAMP is mediated by cGAMP hydrolases or can involve intercellular transfer to propagate immune signals without degradation. Small-molecule inhibitors designed to target either cGAMP degradation or its transport proteins present promising avenues for treating aging, inflammatory, and age-related diseases. ENPP1 inhibits STING signalling by hydrolyzing cGAMP [222], plays a role in the regulation of immune functions [223], and exhibits anti-aging properties by modulating the expression of Klotho, an anti-aging factor [224], under conditions of phosphate overload (Table 1). By regulating extracellular cGAMP, ENPP1 inhibitors enhance STING signalling [225]. They have been employed in antitumor immunotherapy, such as SR-8314 [198–201], MV-626 [198–201], RBS2418 [202, 203], SR-8541A [204], TXN10128 [205, 206], ISM5939 [207, 208] or methotrexate (MTX) [209], and may represent a promising strategy for future anti-aging interventions. SR-8314 and MV-626 are preclinical candidate compounds that have demonstrated significant antitumor activity; however, they have not yet advanced to clinical trials [198–201]. RBS2418 has completed the Phase Ia dose-escalation portion of its clinical trial (NCT05270213), demonstrating favorable safety and target-dependent efficacy [202]. Efficient target engagement was observed across all dose levels. Based on these promising results, a Phase II study (NCT06824064) has been initiated for the treatment of advanced metastatic colorectal cancer [203]. SR-8541A is currently undergoing a Phase I clinical trial (NCT06063681) [204], exhibiting an acceptable safety profile with no dose-limiting toxicities reported to date; all drug-related adverse events have been Grade 1–2. Additionally, a Phase 2 clinical trial (NCT06589440) evaluating SR-8541A in combination with botensilimab and balstilimab for the treatment of refractory metastatic microsatellite-stable colorectal cancer is ongoing [204]. TXN10128 (Txinno Bioscience) represents another ENPP1 inhibitor that has entered Phase I clinical trials (NCT05978492), conducting dose-escalation and expansion studies in patients with advanced solid tumors, with potential combination therapy with irinotecan or paclitaxel [205, 206]. ISM5939 is currently in Phase 1 clinical trials (NCT06724042) for the treatment of advanced or metastatic solid tumors [207, 208]. Low-dose methotrexate (MTX), a first-line therapeutic agent for rheumatoid arthritis, has recently been identified to possess antitumor immunomodulatory activity [209]. MTX binds to the substrate-binding pocket of ENPP1, thereby inhibiting ENPP1-mediated cGAMP hydrolysis and adenosine production, which selectively induces DNA damage in cancer cells, activation of the cGAS-STING pathway, and cGAMP generation. Preliminary clinical investigations have demonstrated that low-dose MTX, in combination with immunotherapy and radiotherapy, exhibits favorable efficacy and safety profiles in patients with unresectable or metastatic solid tumors. Notably, targeting the ENPP1H362A point mutation to block extracellular cGAMP degradation is sufficient to enhance antitumor immunity and antiviral defense, establishing this genetic tool as a specific means to abrogate cGAMP hydrolysis [225]. These insights provide a mechanistic rationale for the development of next-generation ENPP1 inhibitors with substrate-selective activity profiles. Furthermore, recent investigations have revealed that extracellular vesicles derived from apoptotic T cells (ApoEVs) are capable of carrying cGAMP [70]. These vesicles express the surface enzyme ENPP1, which hydrolyzes extracellular cGAMP, thus attenuating the activation of the cGAS-STING signalling pathway and contributing to the alleviation of radiation-induced enteritis [70, 222]. ENPP3 has also been implicated in the promotion of antitumor immunity [55] (Table 1). Recent studies have further uncovered sphingomyelin phosphodiesterase-like 3 A (SMPDL3A), a novel cGAMP-degrading enzyme [56] (Table 1). which specifically inhibits the STING signalling pathway, providing new insights into potential immunotherapeutic approaches for aging-related diseases (Fig. 5). Likewise, SMPDL3B, a GPI-anchored sphingomyelin phosphodiesterase, functions as a membrane integrity sensor that is induced upon viral infection. Its cGAMP hydrolase activity is activated via cGAMP-triggered dimerization, thus serving as a novel negative regulator of cGAS-STING signalling and offering new insights into host–pathogen interactions [226]. Targeting these regulatory enzymes offers a powerful approach to fine-tune cGAMP-mediated immunity, mainly by modulating the canonical STING-dependent pathway.

### Targeting cGAMP transporters

Another promising therapeutic avenue involves modulating the transporters that control cGAMP flux between cells. It is important to note that most therapeutic strategies targeting cGAMP transport ultimately affect STING-dependent signalling, but cGAMP itself can exert both STING-dependent and STING-independent effects depending on the cellular context and concentration. Targeting the principal importers and exporters of extracellular cGAMP has opened a new therapeutic avenue. Pharmacological modulation of these transporters via small molecules or gene-silencing strategies can effectively “gate” intercellular cGAMP traffic, thus tuning the amplitude and duration of STING-dependent immune responses in a tissue-restricted manner.

For instance, sulfasalazine (SSZ), an immunosuppressive drug, directly inhibits the SLC46A2-mediated cGAMP intake [78] (Table 1), a mechanism exploited in the management of inflammatory disorders. The inducers of LL-37, including Vitamin D3 and sodium butyrate, enhance the transmembrane transport of cGAMP, thus effectively triggering the STING signalling pathway [61, 210, 211] (Table 1). These examples illustrate STING-dependent mechanisms where cGAMP functions as the second messenger downstream of cGAS.

A specific inhibitor of MRP1, known as MK-571, has been shown to increase intracellular concentrations of cyclic GMP-AMP (cGAMP) and subsequently activate STING signalling by obstructing the efflux of cGAMP [64] (Table 1). Notably, while the aforementioned transport-modulating strategies mainly enhance STING-dependent immunity, cGAMP can also operate through STING-independent pathways, such as the RAB18-FOSB axis regulating cell migration. This functional dichotomy underscores the importance of context-specific therapeutic design: Strategies aimed at immune activation should target STING-dependent cGAMP signalling, whereas interventions for conditions involving aberrant cell migration might require distinct approaches. Additionally, the inherent carrier capabilities and transmembrane properties of EVs allow for prolonged retention of therapeutics in the circulatory system and enable targeted delivery of drugs, making them a promising vehicle for cGAMP administration [227–229]. Virus-infected cells can release viral particles that transport cGAMP, facilitating its exit from the cell to infect neighboring cells, thus triggering the cytosolic STING pathway and promoting IFN synthesis [96]. Therefore, utilizing EVs [212–214] or viral particles [215] for the delivery of cGAMP as a STING agonist presents a potential avenue for the advancement of immunotherapies aimed at age-related disorders (Table 1). However, the relative contribution of STING-dependent versus STING-independent cGAMP effects in aging remains to be fully elucidated. Furthermore, the utilization of nanoparticles [219, 220, 230], microscale particles [189, 216–218], or liposomes [221] as delivery vehicles for the targeted transportation of cGAMP has emerged as a novel immunotherapy strategy for various STING-associated diseases (Table 1) (Fig. 5).

In conclusion, modulating STING signalling by targeting proteins involved in the synthesis, degradation, and transport of cGAMP may represent an innovative therapeutic approach for addressing various disorders. However, the relative contribution of STING-dependent versus STING-independent cGAMP effects in aging remains to be fully elucidated. The multiplicity of intervention points provides flexibility in designing context-specific therapies.

### As a potential biomarker in diseases

Beyond its therapeutic potential, cGAMP also shows promise as a diagnostic and prognostic biomarker in a variety of diseases. Growing evidence indicates that intracellular or circulating cGAMP levels are significantly associated with disease activity, stage, treatment response, and even long-term prognosis, offering the advantages of quantifiability, standardizability, and suitability for longitudinal monitoring.

In autoimmune diseases such as systemic lupus erythematosus (SLE), rheumatoid arthritis (RA), and psoriasis, plasma/tissue cGAMP levels correlate with disease activity and interferon (IFN) scores [135, 231]. For instance, the median cGAMP concentration in SLE patients during active disease is substantially higher than that in healthy controls. Notably, elevated plasma cGAMP has been observed to precede the appearance of autoantibodies, suggesting its potential as an early molecular predictor for conditions such as Aicardi-Goutières syndrome [231]. Collectively, these observations underscore the potential of cGAMP to serve as a “molecular scale of disease activity.”

In viral infections, cGAMP shows promise as a “host risk index.” In cases of dengue fever with DNA virus co-infection, plasma cGAMP levels exhibit a dose-dependent relationship with the development of dengue shock syndrome (DSS) [232]. Similarly, cGAMP can help predict COVID-19 severity, as critically ill patients demonstrate significantly higher cGAMP levels than those with mild symptoms, and the risk of intubation decreases with declining cGAMP concentrations [233]. In oncology, radiation-induced release of tumor-derived cGAMP activates STING signalling in antigen-presenting cells, promoting CD8⁺ T cell-mediated antitumor immunity and enhanced response to immunotherapy [112]. Moreover, tumor or peripheral blood cGAMP levels correlate with treatment response and may help predict clinical outcomes [113, 117].

In summary, cGAMP has transcended its conventional role as a second messenger and is poised to become a quantitative indicator for assessing disease activity and severity. In cancer, it can preoperatively predict recurrence and enable early evaluation of immunotherapy response. Its performance either surpasses that of traditional inflammatory markers or significantly improves the area under the curve (AUC) when combined with factors such as ENPP1 expression—a key cGAMP hydrolase that attenuates STING signalling [225] —or CD8⁺ T cell infiltration [86]. Furthermore, lowering of cGAMP levels following pharmacological intervention suggests its utility as a potential marker for monitoring therapeutic efficacy. Given the immunomodulatory properties of cGAMP, we suggest that it may represent a potential novel biomarker for clinical applications, offering an objective and quantifiable measure for evaluating early aging indicators. Conventional efficacy assessments typically depend on clinical manifestations or imaging studies, whereas cGAMP provides a more accurate and objective evaluation of treatment outcomes as a molecular biomarker. In cellular therapies for age-related disorders (such as stem cell interventions) [234] or gene therapies [235, 236], cGAMP levels may also help in assessing the restoration of cellular functionality after treatment. However, challenges regarding its stability, delivery efficiency, and immunotoxicity remain, and future research should focus on real-time monitoring and tissue-specific modulation to advance cGAMP-based precision immunotherapeutics.

## Challenges

Despite the considerable promise of cGAMP-based therapeutics and diagnostics, several significant challenges must be addressed before their full potential can be realized. Several obstacles hinder its research and application, as described below:

### cGAMP stability and delivery issues

cGAMP’s instability and susceptibility to enzymatic degradation hinder its research and clinical use [219]. In vitro studies have shown diminished effective concentrations of cGAMP due to degradation, potentially skewing experimental results. Enhancing the stability and efficacy of the delivery of cGAMP poses substantial hurdles in drug development. In order to mitigate adverse effects on healthy tissues, current investigations have focused on strategies to prevent rapid in vivo clearance of cGAMP and to achieve targeted delivery. Overcoming these pharmaceutical challenges is crucial for clinical translation.

### Challenges in the development of cGAMP as a therapeutic agent

Currently, the direct therapeutic use of cGAMP is limited, prompting research into synthetic analogs or small-molecule drugs capable of selectively modulating the STING pathway to minimize off-target effects. Given that STING activation may provoke robust inflammatory responses, prolonged immune activation risks triggering autoimmune disorders or other immune-mediated complications. For instance, in both infection–hypotension and tumor models, cGAMP exhibits a narrow dose–toxicity window; and refractory hypotension and massive T-cell apoptosis rapidly ensue once the dose exceeds a critical range [141, 237–240]. Consequently, the clinical translation of cGAMP must simultaneously overcome the dual hurdles of “robust immune activation” and “toxic cytokine storm”. Future efforts should focus on refined dose- and interval-escalation regimens guided by real-time biomarkers—e.g., plasma inflammatory cytokines, continuous blood-pressure monitoring, dynamic ECG, and multimodal imaging—to maximize therapeutic efficacy while locking toxicities within a controllable margin.

Moreover, current cGAMP-delivery platforms such as liposomes and polymeric nanoparticles universally suffer from low drug-loading capacity, suboptimal membrane-fusion efficiency, and scalability bottlenecks [213, 241–243]. Therefore, advanced materials with superior loading, release, and manufacturing properties are urgently needed to unleash the full therapeutic potential of cGAMP.

In summary, cGAMP plays a highly significant role in terms of immune regulation, anti-infection, antitumor, and senescent cell eradication. Although cGAMP shows promise as a senolytic agent, the long-term effects of sustained immune activation require further investigation. Comprehensive preclinical and clinical investigations are required to evaluate the safety and effectiveness of its prolonged application. With more progress in pharmaceutical development and increased comprehension of the biological mechanisms involving cGAMP, cGAMP may present a promising strategy for managing age-associated diseases in the future. Nonetheless, maintaining an equilibrium between its immunoregulatory impacts and adverse effects, as well as refining the related drug delivery systems and successfully translating these into clinical therapeutics, remain important directions for future research. Looking forward, it is imperative not only to address whether cGAMP can be harnessed, but also to devise strategies for its optimal, affordable, and safe application; and this objective depends on continuous technological refinement, interdisciplinary integration, and stringent evaluative frameworks.

## Conclusion

In conclusion, this review has systematically traced the journey of cGAMP from its molecular synthesis to its complex roles in health and disease. We began by examining the structural and catalytic mechanisms that generate cGAMP’s unique mixed-linkage architecture. Then, we explored the elaborate transport networks—encompassing specific exporters, importers, and vesicular pathways—that enable its function as an intercellular immune messenger. Building on this mechanistic foundation, we analyzed how cGAMP operates across four major disease contexts: As a beneficial defense signal in infectious diseases, a functionally dualistic modulator in cancer, a tolerance-breaking amplifier in autoimmunity, and a driver of chronic inflammation and senescence in aging. These diverse roles informed our discussion of therapeutic strategies targeting cGAMP itself—whether as a vaccine adjuvant, through modulation of its synthesis and degradation, or via manipulation of its transport—and the significant challenges that must be overcome to realize its clinical potential.

Nevertheless, significant knowledge gaps remain. First, the understanding of real-time, dynamic cGAMP transmission in complex in vivo microenvironments, particularly in the context of aging, remains superficial and lacks clear visualization. There is a pressing need to develop advanced tools capable of monitoring extracellular cGAMP concentration and flux in real time and in situ. Second, the cell-type-specific sensitivity to cGAMP and the context-dependent outcomes of its signalling are not fully understood. For instance, why does cGAMP promote antitumor immunity in certain scenarios while driving immunosuppression or tissue fibrosis in others? Furthermore, the pathophysiological significance of newly identified non-canonical signalling pathways, such as the STING-independent RAB18-FOSB axis, in aging and related diseases, and their crosstalk with the canonical IFN signalling constitutes a critical frontier for future exploration. Addressing these fundamental questions will be essential for unlocking the full therapeutic potential of cGAMP modulation and advancing toward precision immunotherapies that harness this remarkable immune messenger.

Over the past decade, cGAMP research has accomplished a “four-stage leap” from a signalling molecule to an immune messenger, then to a disease biomarker, and finally to a therapeutic target. Looking ahead, breakthroughs in this field will rely on interdisciplinary approaches and technological innovation. Future research should prioritize the development of tools for real-time tracking of extracellular cGAMP fluxes, alongside the engineering of delivery systems that enable tissue-specific cGAMP modulation, which would profoundly enhance our functional understanding of this immunotransmitter. For instance, developing genetically encoded fluorescent/bioluminescent cGAMP biosensors will enable dynamic imaging in live organisms. Several biosensors have achieved notable progress to date, exemplified by BioSTING—a FRET-based STING biosensor capable of reporting CDN binding and conformational activity—and RNA-based fluorescent biosensors enabling the detection of cGAMP in cell lysates [244, 245]. These tools collectively facilitate real-time monitoring of cGAMP dynamics and high-throughput screening of cGAS-STING pathway modulators. Coupled with isotope tracing-mass cytometry, these tools can help construct spatially resolved “cGAMP-transcriptome” multi-omics maps. Second, addressing key pharmaceutical challenges—such as low drug-loading capacity, difficulties in scaling up production, and batch-to-batch variability—requires innovative approaches. Utilizing AI-predicted formulations and high-throughput microfluidic synthesis platforms may offer viable solutions. Third, by creating high-affinity cGAMP analogs (“Locks”) through directed evolution or chemical synthesis and developing programmable proteases or ribozymes (“Switches”), we could establish molecular systems capable of millisecond-level “on–off” control, thus enabling precise immune modulation. Finally, by integrating machine learning with multi-scale modeling, it will help predict cGAMP dose–response relationships across diverse tissue microenvironments. By implementing digital twin technology, we could simulate personalized dosing strategies in silico, significantly reducing experimental trial-and-error and accelerating translational applications. At the translational level, the focus should shift towards developing next-generation cGAMP-targeting strategies, including: (1) designing tissue-targeted nanodelivery systems for cGAMP to maximize efficacy and minimize systemic toxicity; (2) exploring combination therapies that concurrently regulate its synthesis (cGAS), degradation (ENPP1 and SMPDL3A), and transport (ABCC1 and SLC19A1); and (3) conducting rigorous clinical studies to validate the feasibility of cGAMP as a diagnostic biomarker for diseases. These multidisciplinary efforts will be crucial for translating our growing knowledge of cGAMP biology into meaningful clinical advances.

In summary, research focusing on cGAMP not only deepens our understanding of innate immunity and cellular communication but also unveils a promising new therapeutic paradigm. By achieving precise manipulation of this “immune messenger,” we can anticipate the development of more targeted, effective, and safe immunotherapeutic strategies to address a spectrum of major health challenges, including infections, cancer, autoimmune diseases, and aging-associated disorders in the future. As we continue to unravel the complexities of cGAMP biology, we move closer to harnessing its potential for improving human health and combating age-related decline, ultimately paving the way for a new era in immunomodulatory medicine.

## Data Availability

The authors declare that all data presented in this study will be presented upon request from the corresponding author.

## References

[1] Hopfner KP, Hornung V. Molecular mechanisms and cellular functions of cGAS-STING signalling. Nat Rev Mol Cell Biol. 2020;21(9):501–21. 10.1038/s41580-020-0244-x. 32424334 10.1038/s41580-020-0244-x

[2] Patel DJ, Yu Y, Xie W. cGAMP-activated cGAS-STING signaling: its bacterial origins and evolutionary adaptation by metazoans. Nat Struct Mol Biol. 2023;30(3):245–60. 10.1038/s41594-023-00933-9. 36894694 10.1038/s41594-023-00933-9 PMC11749898

[3] Ritchie C, Carozza JA, Li L. Biochemistry, cell biology, and pathophysiology of the innate immune cGAS-cGAMP-STING pathway. Annu Rev Biochem. 2022;91:599–628. 10.1146/annurev-biochem-040320-101629. 35287475 10.1146/annurev-biochem-040320-101629

[4] Chen C, Xu P. Cellular functions of cGAS-STING signaling. Trends Cell Biol. 2023;33(8):630–48. 10.1016/j.tcb.2022.11.001. 36437149 10.1016/j.tcb.2022.11.001

[5] Sun Z, Hornung V. cGAS-STING signaling. Curr Biol. 2022;32(13):R730–4. 10.1016/j.cub.2022.05.027. 35820380 10.1016/j.cub.2022.05.027

[6] Lazear HM, Schoggins JW, Diamond MS. Shared and distinct functions of type I and type III interferons. Immunity. 2019;50(4):907–23. 10.1016/j.immuni.2019.03.025. 30995506 10.1016/j.immuni.2019.03.025 PMC6839410

[7] Frisch SM, MacFawn IP. Type I interferons and related pathways in cell senescence. Aging Cell. 2020;19(10):e13234. 10.1111/acel.13234. 32918364 10.1111/acel.13234 PMC7576263

[8] Snell LM, McGaha TL, Brooks DG. Type I interferon in chronic virus infection and cancer. Trends Immunol. 2017;38(8):542–57. 10.1016/j.it.2017.05.005. 28579323 10.1016/j.it.2017.05.005 PMC8059441

[9] Zitvogel L, Galluzzi L, Kepp O, Smyth MJ, Kroemer G. Type I interferons in anticancer immunity. Nat Rev Immunol. 2015;15(7):405–14. 10.1038/nri3845. 26027717 10.1038/nri3845

[10] Mesev EV, LeDesma RA, Ploss A. Decoding type I and III interferon signalling during viral infection. Nat Microbiol. 2019;4(6):914–24. 10.1038/s41564-019-0421-x. 30936491 10.1038/s41564-019-0421-x PMC6554024

[11] Peignier A, Parker D. Impact of Type I interferons on susceptibility to bacterial pathogens. Trends Microbiol. 2021;29(9):823–35. 10.1016/j.tim.2021.01.007. 33546974 10.1016/j.tim.2021.01.007 PMC8326292

[12] Gao M, He Y, Tang H, Chen X, Liu S, Tao Y. cGAS/STING: novel perspectives of the classic pathway. Mol Biomed. 2020;1(1):7. 10.1186/s43556-020-00006-z. 35006429 10.1186/s43556-020-00006-z PMC8603984

[13] Sun X, Jia X, Lu Z, Tang J, Li M. Drug repositioning with adaptive graph convolutional networks. Bioinformatics. 2024. 10.1093/bioinformatics/btad748. 38070161 10.1093/bioinformatics/btad748 PMC10761094

[14] Schmitz CRR, Maurmann RM, Guma F, Bauer ME, Barbe-Tuana FM. cGAS-STING pathway as a potential trigger of immunosenescence and inflammaging. Front Immunol. 2023;14:1132653. 10.3389/fimmu.2023.1132653. 36926349 10.3389/fimmu.2023.1132653 PMC10011111

[15] Dvorkin S, Cambier S, Volkman HE, Stetson DB. New frontiers in the cGAS-STING intracellular DNA-sensing pathway. Immunity. 2024;57(4):718–30. 10.1016/j.immuni.2024.02.019. 38599167 10.1016/j.immuni.2024.02.019 PMC11013568

[16] Hao X, Zhao B, Towers M, Liao L, Monteiro EL, Xu X, et al. TXNRD1 drives the innate immune response in senescent cells with implications for age-associated inflammation. Nat Aging. 2024;4(2):185–97. 10.1038/s43587-023-00564-1. 38267705 10.1038/s43587-023-00564-1 PMC11210448

[17] Herbstein F, Sapochnik M, Attorresi A, Pollak C, Senin S, Gonilski-Pacin D, et al. The SASP factor IL-6 sustains cell-autonomous senescent cells via a cGAS-STING-NFkappaB intracrine senescent noncanonical pathway. Aging Cell. 2024;23(10):e14258. 10.1111/acel.14258. 39012326 10.1111/acel.14258 PMC11464112

[18] Victorelli S, Salmonowicz H, Chapman J, Martini H, Vizioli MG, Riley JS, et al. Apoptotic stress causes mtDNA release during senescence and drives the SASP. Nature. 2023;622(7983):627–36. 10.1038/s41586-023-06621-4. 37821702 10.1038/s41586-023-06621-4 PMC10584674

[19] Hao X, Wang C, Zhang R. Chromatin basis of the senescence-associated secretory phenotype. Trends Cell Biol. 2022;32(6):513–26. 10.1016/j.tcb.2021.12.003. 35012849 10.1016/j.tcb.2021.12.003 PMC9106822

[20] Lucini CB, Braun RJ. Mitochondrion-dependent cell death in TDP-43 proteinopathies. Biomedicines. 2021. 10.3390/biomedicines9040376. 33918437 10.3390/biomedicines9040376 PMC8066287

[21] Blest HTW, Chauveau L. cGAMP the travelling messenger. Front Immunol. 2023;14:1150705. 10.3389/fimmu.2023.1150705. 37287967 10.3389/fimmu.2023.1150705 PMC10242147

[22] Ma XY, Chen MM, Meng LH. Second messenger 2’3’-cyclic GMP-AMP (2’3’-cGAMP): the cell autonomous and non-autonomous roles in cancer progression. Acta Pharmacol Sin. 2024;45(5):890–9. 10.1038/s41401-023-01210-7. 38177693 10.1038/s41401-023-01210-7 PMC11053103

[23] Wang L, Li S, Wang K, Wang N, Liu Q, Sun Z, et al. DNA mechanical flexibility controls DNA potential to activate cGAS-mediated immune surveillance. Nat Commun. 2022;13(1):7107. 10.1038/s41467-022-34858-6. 36402783 10.1038/s41467-022-34858-6 PMC9675814

[24] Hertzog J, Rehwinkel J. Regulation and inhibition of the DNA sensor cGAS. EMBO Rep. 2020;21(12):e51345. 10.15252/embr.202051345. 33155371 10.15252/embr.202051345 PMC7726805

[25] Liu Y, Xu P. cGAS, an innate dsDNA sensor with multifaceted functions. Cell Insight. 2025;4(3):100249. 10.1016/j.cellin.2025.100249. 40470467 10.1016/j.cellin.2025.100249 PMC12135389

[26] Li T, Huang T, Du M, Chen X, Du F, Ren J, et al. Phosphorylation and chromatin tethering prevent cGAS activation during mitosis. Science. 2021. 10.1126/science.abc5386. 33542149 10.1126/science.abc5386 PMC8171060

[27] Kujirai T, Zierhut C, Takizawa Y, Kim R, Negishi L, Uruma N, et al. Structural basis for the inhibition of cGAS by nucleosomes. Science. 2020;370(6515):455–8. 10.1126/science.abd0237. 32912999 10.1126/science.abd0237 PMC7584773

[28] Hornung V, Hartmann R, Ablasser A, Hopfner KP. OAS proteins and cGAS: Unifying concepts in sensing and responding to cytosolic nucleic acids. Nat Rev Immunol. 2014;14(8):521–8. 10.1038/nri3719. 25033909 10.1038/nri3719 PMC7097587

[29] Zhang Z, Zhang C. Regulation of cGAS-STING signalling and its diversity of cellular outcomes. Nat Rev Immunol. 2025;25(6):425–44. 10.1038/s41577-024-01112-7. 39774812 10.1038/s41577-024-01112-7

[30] Zhang B, Xu P, Ablasser A. Regulation of the cGAS-STING pathway. Annu Rev Immunol. 2025;43(1):667–92. 10.1146/annurev-immunol-101721-032910. 40085836 10.1146/annurev-immunol-101721-032910

[31] Zhang X, Wu J, Du F, Xu H, Sun L, Chen Z, et al. The cytosolic DNA sensor cGAS forms an oligomeric complex with DNA and undergoes switch-like conformational changes in the activation loop. Cell Rep. 2014;6(3):421–30. 10.1016/j.celrep.2014.01.003. 24462292 10.1016/j.celrep.2014.01.003 PMC3969844

[32] Boyer JA, Spangler CJ, Strauss JD, Cesmat AP, Liu P, McGinty RK, et al. Structural basis of nucleosome-dependent cGAS inhibition. Science. 2020;370(6515):450–4. 10.1126/science.abd0609. 32913000 10.1126/science.abd0609 PMC8189757

[33] Zhou W, Mohr L, Maciejowski J, Kranzusch PJ. cGAS phase separation inhibits TREX1-mediated DNA degradation and enhances cytosolic DNA sensing. Mol Cell. 2021;81(4):739-55 e7. 10.1016/j.molcel.2021.01.024. 33606975 10.1016/j.molcel.2021.01.024 PMC7899126

[34] Hall J, Ralph EC, Shanker S, Wang H, Byrnes LJ, Horst R, et al. The catalytic mechanism of cyclic GMP-AMP synthase (cGAS) and implications for innate immunity and inhibition. Protein Sci. 2017;26(12):2367–80. 10.1002/pro.3304. 28940468 10.1002/pro.3304 PMC5699495

[35] Wu S, Gabelli SB, Sohn J. The structural basis for 2’-5’/3’-5’-cGAMP synthesis by cGAS. Nat Commun. 2024;15(1):4012. 10.1038/s41467-024-48365-3. 38740774 10.1038/s41467-024-48365-3 PMC11091121

[36] Zhao Z, Ma Z, Wang B, Guan Y, Su XD, Jiang Z. Mn(2+) directly activates cGAS and structural analysis suggests Mn(2+) induces a noncanonical catalytic synthesis of 2’3’-cGAMP. Cell Rep. 2020;32(7):108053. 10.1016/j.celrep.2020.108053. 32814054 10.1016/j.celrep.2020.108053

[37] Kulkarni R, Maranholkar V, Nguyen N, Cirino PC, Willson RC, Varadarajan N. The efficient synthesis and purification of 2’3’- cGAMP from *Escherichia coli*. Front Microbiol. 2024;15:1345617. 10.3389/fmicb.2024.1345617. 38525075 10.3389/fmicb.2024.1345617 PMC10957790

[38] Ablasser A, Goldeck M, Cavlar T, Deimling T, Witte G, Rohl I, et al. cGAS produces a 2’-5’-linked cyclic dinucleotide second messenger that activates STING. Nature. 2013;498(7454):380–4. 10.1038/nature12306. 23722158 10.1038/nature12306 PMC4143541

[39] Gao P, Ascano M, Wu Y, Barchet W, Gaffney BL, Zillinger T, et al. Cyclic [G(2’,5’)pA(3’,5’)p] is the metazoan second messenger produced by DNA-activated cyclic GMP-AMP synthase. Cell. 2013;153(5):1094–107. 10.1016/j.cell.2013.04.046. 23647843 10.1016/j.cell.2013.04.046 PMC4382009

[40] Liu C, Shi R, Jensen MS, Zhu J, Liu J, Liu X, et al. The global regulation of c-di-GMP and cAMP in bacteria. mLife. 2024;3(1):42–56. 10.1002/mlf2.12104. 38827514 10.1002/mlf2.12104 PMC11139211

[41] Hussain B, Xie Y, Jabeen U, Lu D, Yang B, Wu C, et al. Activation of STING based on its structural features. Front Immunol. 2022;13:808607. 10.3389/fimmu.2022.808607. 35928815 10.3389/fimmu.2022.808607 PMC9343627

[42] Klima M, Dejmek M, Duchoslav V, Eisenreichova A, Sala M, Chalupsky K, et al. Fluorinated cGAMP analogs, which act as STING agonists and are not cleavable by poxins: structural basis of their function. Structure. 2024;32(4):433-9 e4. 10.1016/j.str.2024.01.008. 38325369 10.1016/j.str.2024.01.008

[43] Shen M, Jiang X, Peng Q, Oyang L, Ren Z, Wang J, et al. The cGAS‒STING pathway in cancer immunity: mechanisms, challenges, and therapeutic implications. J Hematol Oncol. 2025;18(1):40. 10.1186/s13045-025-01691-5. 40188340 10.1186/s13045-025-01691-5 PMC11972543

[44] Pu F, Chen F, Liu J, Zhang Z, Shao Z. Immune regulation of the cGAS-STING signaling pathway in the tumor microenvironment and its clinical application. Onco Targets Ther. 2021;14:1501–16. 10.2147/OTT.S298958. 33688199 10.2147/OTT.S298958 PMC7935450

[45] Yu Y, Liu J, Liu C, Liu R, Liu L, Yu Z, et al. Post-translational modifications of cGAS-STING: a critical switch for immune regulation. Cells. 2022. 10.3390/cells11193043. 36231006 10.3390/cells11193043 PMC9563579

[46] Lu Y, Zhao M, Chen L, Wang Y, Liu T, Liu H. cGAS: action in the nucleus. Front Immunol. 2024;15:1380517. 10.3389/fimmu.2024.1380517. 38515746 10.3389/fimmu.2024.1380517 PMC10954897

[47] Wang M, Fan B, Lu W, Ryde U, Chang Y, Han D, et al. Unraveling the binding mode of cyclic adenosine-inosine monophosphate (cAIMP) to STING through molecular dynamics simulations. Molecules. 2024. 10.3390/molecules29112650. 38893524 10.3390/molecules29112650 PMC11173896

[48] Li B, Zhang C, Xu X, Shen Q, Luo S, Hu J. Manipulating the cGAS-STING axis: advancing innovative strategies for osteosarcoma therapeutics. Front Immunol. 2025;16:1539396. 10.3389/fimmu.2025.1539396. 39991153 10.3389/fimmu.2025.1539396 PMC11842356

[49] Geelen D, Beacom A, Vaurs M, Mahieu M, Boutoual R, Derumier A et al. Constitutive cGAS-STING activation in ALT+ cells. 2025:2025.04.18.649516. 10.1101/2025.04.18.649516 %J bioRxiv.

[50] Xie W, Lama L, Yang X, Kuryavyi V, Bhattacharya S, Nudelman I et al. Arabinose- and xylose-modified analogs of 2',3'-cGAMP act as STING agonists. Cell Chem Biol. 2023;30(11):1366–76 e7. 10.1016/j.chembiol.2023.07.002. 10.1016/j.chembiol.2023.07.002 PMC10808274 37536341

[51] Lu Y, You L, Li L, Kilgore JA, Liu S, Wang X, et al. Orthogonal hydroxyl functionalization of cGAMP confers metabolic stability and enables antibody conjugation. ACS Cent Sci. 2023;9(12):2298–305. 10.1021/acscentsci.3c01122. 38161369 10.1021/acscentsci.3c01122 PMC10755847

[52] Sun P, Wang B, Liu C, Wang Z, Liu Y, Qiao YB, et al. A fluorescent STING ligand sensor for high-throughput screening of compounds that can enhance tumor immunotherapy. Cell Rep Methods. 2025;5(7):101106. 10.1016/j.crmeth.2025.101106. 40669456 10.1016/j.crmeth.2025.101106 PMC12296492

[53] Li L, He Y, Chen Y, Zhou X. cGAS-STING pathway’s impact on intestinal barrier. J Gastroenterol Hepatol. 2025;40(6):1381–92. 10.1111/jgh.16974. 40377214 10.1111/jgh.16974

[54] Kato K, Nishimasu H, Oikawa D, Hirano S, Hirano H, Kasuya G, et al. Structural insights into cGAMP degradation by ecto-nucleotide pyrophosphatase phosphodiesterase 1. Nat Commun. 2018;9(1):4424. 10.1038/s41467-018-06922-7. 30356045 10.1038/s41467-018-06922-7 PMC6200793

[55] Mardjuki R, Wang S, Carozza JA, Abhiraman GC, Lyu X, Li L. Identification of extracellular membrane protein ENPP3 as a major cGAMP hydrolase, cementing cGAMP's role as an immunotransmitter. bioRxiv. 2024. 10.1101/2024.01.12.575449.

[56] Hou Y, Wang Z, Liu P, Wei X, Zhang Z, Fan S et al. SMPDL3A is a cGAMP-degrading enzyme induced by LXR-mediated lipid metabolism to restrict cGAS-STING DNA sensing. Immunity. 2023;56(11):2492–507 e10. 10.1016/j.immuni.2023.10.001. 10.1016/j.immuni.2023.10.001 37890481

[57] Li L, Yin Q, Kuss P, Maliga Z, Millan JL, Wu H, et al. Hydrolysis of 2’3’-cGAMP by ENPP1 and design of nonhydrolyzable analogs. Nat Chem Biol. 2014;10(12):1043–8. 10.1038/nchembio.1661. 25344812 10.1038/nchembio.1661 PMC4232468

[58] Mardjuki R, Wang S, Carozza J, Zirak B, Subramanyam V, Abhiraman G, et al. Identification of the extracellular membrane protein ENPP3 as a major cGAMP hydrolase and innate immune checkpoint. Cell Rep. 2024;43(5):114209. 10.1016/j.celrep.2024.114209. 38749434 10.1016/j.celrep.2024.114209 PMC13268100

[59] Shin H, Chung H. SMPDL3A links cholesterol metabolism to the cGAS-STING pathway. Immunity. 2023;56(11):2459–61. 10.1016/j.immuni.2023.10.015. 37967525 10.1016/j.immuni.2023.10.015 PMC11056274

[60] Xie W, Patel DJ. Structure-based mechanisms of 2’3’-cGAMP intercellular transport in the cGAS-STING immune pathway. Trends Immunol. 2023;44(6):450–67. 10.1016/j.it.2023.04.006. 37147228 10.1016/j.it.2023.04.006 PMC11824902

[61] Wei X, Zhang L, Yang Y, Hou Y, Xu Y, Wang Z, et al. LL-37 transports immunoreactive cGAMP to activate STING signaling and enhance interferon-mediated host antiviral immunity. Cell Rep. 2022;39(9):110880. 10.1016/j.celrep.2022.110880. 35649354 10.1016/j.celrep.2022.110880

[62] Dane EL, Belessiotis-Richards A, Backlund C, Wang J, Hidaka K, Milling LE, et al. STING agonist delivery by tumour-penetrating PEG-lipid nanodiscs primes robust anticancer immunity. Nat Mater. 2022;21(6):710–20. 10.1038/s41563-022-01251-z. 35606429 10.1038/s41563-022-01251-z PMC9156412

[63] Shinde O, Boyer JA, Cambier S, VanPortfliet JJ, Sui X, Yadav GP et al. Structures of ATP-binding cassette transporter ABCC1 reveal the molecular basis of cyclic dinucleotide cGAMP export. Immunity. 2025;58(1):59–73 e5. 10.1016/j.immuni.2024.12.002. 10.1016/j.immuni.2024.12.002 PMC11735300 39765229

[64] Maltbaek JH, Cambier S, Snyder JM, Stetson DB. ABCC1 transporter exports the immunostimulatory cyclic dinucleotide cGAMP. Immunity. 2022;55(10):1799–812 e4. 10.1016/j.immuni.2022.08.006. 10.1016/j.immuni.2022.08.006 PMC9561016 36070769

[65] Wang Q, Yu Y, Zhuang J, Liu R, Sun C. Demystifying the cGAS-STING pathway: precision regulation in the tumor immune microenvironment. Mol Cancer. 2025;24(1):178. 10.1186/s12943-025-02380-0. 40506729 10.1186/s12943-025-02380-0 PMC12160120

[66] Zhang Z, Gao J, Liao X, Zhang Z, Cao X, Gong Y, et al. ABCC10-mediated cGAMP efflux drives cancer cell radiotherapy resistance. Cell Death Differ. 2025. 10.1038/s41418-025-01552-1. 40770563 10.1038/s41418-025-01552-1 PMC12811381

[67] Szemere ZK, Murphy EA. Import of extracellular 2’-3’cGAMP by the folate transporter, SLC19A1, establishes an antiviral response that limits herpes simplex virus-1. Antivir Res. 2024;230:105989. 10.1016/j.antiviral.2024.105989. 39154753 10.1016/j.antiviral.2024.105989 PMC11827581

[68] Xiao L, Ai YL, Mi XY, Liang H, Zhi X, Wu LZ et al. cGAS activation converges with intracellular acidification to promote STING aggregation and pyroptosis in tumor models. J Clin Invest. 2025;135(18). 10.1172/JCI188872. 10.1172/JCI188872 PMC12435844 40663398

[69] Wang M, Xu P, Wu Q. Cell-to-cell communications of cGAS-STING pathway in tumor immune microenvironment. Zhejiang Da Xue Xue Bao Yi Xue Ban. 2024;53(1):15–24. 10.3724/zdxbyxb-2023-0482. 38229499 10.3724/zdxbyxb-2023-0482 PMC10945497

[70] Zhou Y, Bao L, Gong S, Dou G, Li Z, Wang Z, et al. T cell-derived apoptotic extracellular vesicles hydrolyze cGAMP to alleviate radiation enteritis via surface enzyme ENPP1. Adv Sci. 2024;11(31):e2401634. 10.1002/advs.202401634. 10.1002/advs.202401634 PMC11336903 38888507

[71] Kadota T, Fujita Y, Yoshioka Y, Araya J, Kuwano K, Ochiya T. Emerging role of extracellular vesicles as a senescence-associated secretory phenotype: insights into the pathophysiology of lung diseases. Mol Aspects Med. 2018;60:92–103. 10.1016/j.mam.2017.11.005. 29146100 10.1016/j.mam.2017.11.005

[72] Yin Y, Chen H, Wang Y, Zhang L, Wang X. Roles of extracellular vesicles in the aging microenvironment and age-related diseases. J Extracell Vesicles. 2021;10(12):e12154. 10.1002/jev2.12154. 34609061 10.1002/jev2.12154 PMC8491204

[73] Du S, Ling H, Guo Z, Cao Q, Song C. Roles of exosomal miRNA in vascular aging. Pharmacol Res. 2021;165:105278. 10.1016/j.phrs.2020.105278. 33166733 10.1016/j.phrs.2020.105278

[74] Liang J, Yin H. STAM transports STING oligomers into extracellular vesicles, down-regulating the innate immune response. J Extracell Vesicles. 2023;12(3):e12316. 10.1002/jev2.12316. 36946680 10.1002/jev2.12316 PMC10032202

[75] Jin J, Mu Y, Zhang H, Sturmlechner I, Wang C, Jadhav RR, et al. CISH impairs lysosomal function in activated T cells resulting in mitochondrial DNA release and inflammaging. Nat Aging. 2023;3(5):600–16. 10.1038/s43587-023-00399-w. 37118554 10.1038/s43587-023-00399-w PMC10388378

[76] Ritchie C, Cordova AF, Hess GT, Bassik MC, Li L. SLC19A1 Is an Importer of the Immunotransmitter cGAMP. Mol Cell. 2019;75(2):372–81 e5. 10.1016/j.molcel.2019.05.006. 10.1016/j.molcel.2019.05.006 PMC6711396 31126740

[77] Zhang Q, Zhang X, Liu K, Zhu Y, Nie X, Ma J, et al. Molecular basis of SLC19A1-mediated folate and cyclic dinucleotide transport. Nat Commun. 2025;16(1):3146. 10.1038/s41467-025-58378-1. 40175380 10.1038/s41467-025-58378-1 PMC11965291

[78] Cordova AF, Ritchie C, Bohnert V, Li L. Human SLC46A2 is the dominant cGAMP importer in extracellular cGAMP-sensing macrophages and monocytes. ACS Cent Sci. 2021;7(6):1073–88. 10.1021/acscentsci.1c00440. 34235268 10.1021/acscentsci.1c00440 PMC8228594

[79] Zhou Y, Fei M, Zhang G, Liang WC, Lin W, Wu Y et al. Blockade of the Phagocytic Receptor MerTK on Tumor-Associated Macrophages Enhances P2X7R-Dependent STING Activation by Tumor-Derived cGAMP. Immunity. 2020;52(2):357–73 e9. 10.1016/j.immuni.2020.01.014. 10.1016/j.immuni.2020.01.014 32049051

[80] Ge X, Zhu X, Liu W, Li M, Zhang Z, Zou M, et al. cGAMP promotes inner blood-retinal barrier breakdown through P2RX7-mediated transportation into microglia. J Neuroinflammation. 2025;22(1):58. 10.1186/s12974-025-03391-w. 40025497 10.1186/s12974-025-03391-w PMC11871612

[81] North RA. Molecular physiology of P2X receptors. Physiol Rev. 2002;82(4):1013–67. 10.1152/physrev.00015.2002. 12270951 10.1152/physrev.00015.2002

[82] Wu Q, Leng X, Xu P. Intercellular transmission of cGAS-STING signaling in cancer. Cancer Biol Med. 2023;20(2):93–7. 10.20892/j.issn.2095-3941.2022.0750. 36861445 10.20892/j.issn.2095-3941.2022.0750 PMC9978895

[83] Zhou C, Chen X, Planells-Cases R, Chu J, Wang L, Cao L et al. Transfer of cGAMP into Bystander Cells via LRRC8 Volume-Regulated Anion Channels Augments STING-Mediated Interferon Responses and Anti-viral Immunity. Immunity. 2020;52(5):767–81 e6. 10.1016/j.immuni.2020.03.016. 10.1016/j.immuni.2020.03.016 32277911

[84] Wang L, Cao L, Li Z, Shao Z, Chen X, Huang Z, et al. ATP-elicited cation fluxes promote volume-regulated anion channel LRRC8/VRAC transport cGAMP for antitumor immunity. J Immunol. 2024;213(3):347–61. 10.4049/jimmunol.2300812. 38847616 10.4049/jimmunol.2300812

[85] Yoon J, Kim S, Lee M, Kim Y. Mitochondrial nucleic acids in innate immunity and beyond. Exp Mol Med. 2023;55(12):2508–18. 10.1038/s12276-023-01121-x. 38036728 10.1038/s12276-023-01121-x PMC10766607

[86] Carozza JA, Bohnert V, Nguyen KC, Skariah G, Shaw KE, Brown JA, et al. Extracellular cGAMP is a cancer cell-produced immunotransmitter involved in radiation-induced anti-cancer immunity. Nat Cancer. 2020;1(2):184–96. 10.1038/s43018-020-0028-4. 33768207 10.1038/s43018-020-0028-4 PMC7990037

[87] Wang J, Wang X, Xiong Q, Gao S, Wang S, Zhu S, et al. A dual-STING-activating nanosystem expands cancer immunotherapeutic temporal window. Cell Rep Med. 2024;5(11):101797. 10.1016/j.xcrm.2024.101797. 39454571 10.1016/j.xcrm.2024.101797 PMC11604482

[88] Zhou L, Ho BM, Chan HYE, Tong Y, Du L, He JN, et al. Emerging roles of cGAS-STING signaling in mediating ocular inflammation. J Innate Immun. 2023;15(1):739–50. 10.1159/000533897. 37778330 10.1159/000533897 PMC10616671

[89] Passarella S, Kethiswaran S, Brandes K, Tsai IC, Cebulski K, Kroger A, et al. Alteration of cGAS-STING signaling pathway components in the mouse cortex and hippocampus during healthy brain aging. Front Aging Neurosci. 2024;16:1429005. 10.3389/fnagi.2024.1429005. 39149145 10.3389/fnagi.2024.1429005 PMC11324507

[90] Chung S, Jeong JH, Park JC, Han JW, Lee Y, Kim JI, et al. Blockade of STING activation alleviates microglial dysfunction and a broad spectrum of Alzheimer’s disease pathologies. Exp Mol Med. 2024;56(9):1936–51. 10.1038/s12276-024-01295-y. 39218977 10.1038/s12276-024-01295-y PMC11447230

[91] Skopelja-Gardner S, An J, Elkon KB. Role of the cGAS-STING pathway in systemic and organ-specific diseases. Nat Rev Nephrol. 2022;18(9):558–72. 10.1038/s41581-022-00589-6. 35732833 10.1038/s41581-022-00589-6 PMC9214686

[92] Carozza JA, Cordova AF, AlSaif Y, Böhnert V, Skariah G, Li L. Probing pathophysiology of extracellular cGAMP with substrate-selective ENPP1. 2021:2021.05.04.442665. 10.1101/2021.05.04.442665 %J bioRxiv.

[93] Jiang S, Xia N, Luo J, Zhang Y, Cao Q, Zhang J, et al. The porcine cyclic GMP-AMP synthase-STING pathway exerts an unusual antiviral function independent of interferon and autophagy. J Virol. 2022;96(23):e0147622. 10.1128/jvi.01476-22. 36377876 10.1128/jvi.01476-22 PMC9749457

[94] Cheng Z, Dai T, He X, Zhang Z, Xie F, Wang S, et al. The interactions between cGAS-STING pathway and pathogens. Signal Transduct Target Ther. 2020;5(1):91. 10.1038/s41392-020-0198-7. 32532954 10.1038/s41392-020-0198-7 PMC7293265

[95] Gentili M, Kowal J, Tkach M, Satoh T, Lahaye X, Conrad C, et al. Transmission of innate immune signaling by packaging of cGAMP in viral particles. Science. 2015;349(6253):1232–6. 10.1126/science.aab3628. 26229115 10.1126/science.aab3628

[96] Bridgeman A, Maelfait J, Davenne T, Partridge T, Peng Y, Mayer A, et al. Viruses transfer the antiviral second messenger cGAMP between cells. Science. 2015;349(6253):1228–32. 10.1126/science.aab3632. 26229117 10.1126/science.aab3632 PMC4617605

[97] Pepin G, De Nardo D, Rootes CL, Ullah TR, Al-Asmari SS, Balka KR et al. Connexin-Dependent Transfer of cGAMP to Phagocytes Modulates Antiviral Responses. mBio. 2020;11(1). 10.1128/mBio.03187-19. 10.1128/mBio.03187-19 PMC6989113 31992625

[98] Liu X, Zhang H, Xu L, Ye H, Huang J, Jing X, et al. cGAMP-targeting injectable hydrogel system promotes periodontal restoration by alleviating cGAS-STING pathway activation. Bioact Mater. 2025;48:55–70. 10.1016/j.bioactmat.2025.02.010. 40303968 10.1016/j.bioactmat.2025.02.010 PMC12038443

[99] Guo X, Shu C, Li H, Pei Y, Woo SL, Zheng J, et al. Cyclic GMP-AMP ameliorates diet-induced metabolic dysregulation and regulates proinflammatory responses distinctly from STING activation. Sci Rep. 2017;7(1):6355. 10.1038/s41598-017-05884-y. 28743914 10.1038/s41598-017-05884-y PMC5526935

[100] Zhang D, Liu Y, Zhu Y, Zhang Q, Guan H, Liu S, et al. A non-canonical cGAS-STING-PERK pathway facilitates the translational program critical for senescence and organ fibrosis. Nat Cell Biol. 2022;24(5):766–82. 10.1038/s41556-022-00894-z. 35501370 10.1038/s41556-022-00894-z

[101] Zhang Q, Ding H, Dai Z, Yang R, Zhou S, Tai S. U-shaped association between plasma cyclic guanosine monophosphate-adenosine monophosphate (cGAMP) levels and myocardial infarction. BMC Cardiovasc Disord. 2025;25(1):116. 10.1186/s12872-025-04543-9. 39972291 10.1186/s12872-025-04543-9 PMC11837390

[102] Chen R, Du J, Zhu H, Ling Q. The role of cGAS-STING signalling in liver diseases. JHEP Rep. 2021;3(5):100324. 10.1016/j.jhepr.2021.100324. 34381984 10.1016/j.jhepr.2021.100324 PMC8340306

[103] Wei XY, Gong XJ, Ji H. Research progress on the cGAS-STING signaling pathway in immune-mediated inflammatory diseases in children. Zhongguo Dang Dai Er Ke Za Zhi. 2025;27(7):881–7. 10.7499/j.issn.1008-8830.2412098. 40695524 10.7499/j.issn.1008-8830.2412098 PMC12291564

[104] Gong J, Gao X, Ge S, Li H, Wang R, Zhao L. The role of cGAS-STING signalling in metabolic diseases: from signalling networks to targeted intervention. Int J Biol Sci. 2024;20(1):152–74. 10.7150/ijbs.84890. 38164186 10.7150/ijbs.84890 PMC10750282

[105] Gulen MF, Samson N, Keller A, Schwabenland M, Liu C, Gluck S, et al. cGAS-STING drives ageing-related inflammation and neurodegeneration. Nature. 2023;620(7973):374–80. 10.1038/s41586-023-06373-1. 37532932 10.1038/s41586-023-06373-1 PMC10412454

[106] S Anwar KU Islam I Azmi J Iqbal. Activation of cGAS-STING signaling pathway during HCV infection bioRxiv. 2025 632524 10.1101/2025.01.11.632524

[107] Zhu Q, Hu H, Liu H, Shen H, Yan Z, Gao L. A synthetic STING agonist inhibits the replication of human parainfluenza virus 3 and rhinovirus 16 through distinct mechanisms. Antivir Res. 2020;183:104933. 10.1016/j.antiviral.2020.104933. 32949635 10.1016/j.antiviral.2020.104933 PMC7494516

[108] Yang B, Hu A, Wang T, Chen X, Ma C, Yang X, et al. SARS-CoV-2 infection induces ZBP1-dependent PANoptosis in bystander cells. Proc Natl Acad Sci U S A. 2025;122(28):e2500208122. 10.1073/pnas.2500208122. 40627395 10.1073/pnas.2500208122 PMC12280982

[109] Xu S, Ducroux A, Ponnurangam A, Vieyres G, Franz S, Musken M, et al. cGAS-mediated innate immunity spreads intercellularly through HIV-1 Env-induced membrane fusion sites. Cell Host Microbe. 2016;20(4):443–57. 10.1016/j.chom.2016.09.003. 27736643 10.1016/j.chom.2016.09.003

[110] Mertens RT, Misra A, Xiao P, Baek S, Rone JM, Mangani D, et al. A metabolic switch orchestrated by IL-18 and the cyclic dinucleotide cGAMP programs intestinal tolerance. Immunity. 2024;57(9):2077-94 e12. 10.1016/j.immuni.2024.06.001. 38906145 10.1016/j.immuni.2024.06.001

[111] Su J, Coleman P, Ntorla A, Anderson R, Shattock MJ, Burgoyne JR. Sensing cytosolic DNA lowers blood pressure by direct cGAMP-dependent PKGI activation. Circulation. 2023;148(13):1023–34. 10.1161/CIRCULATIONAHA.123.065547. 37548012 10.1161/CIRCULATIONAHA.123.065547 PMC10516174

[112] Du JM, Qian MJ, Yuan T, Chen RH, He QJ, Yang B, et al. cGAS and cancer therapy: a double-edged sword. Acta Pharmacol Sin. 2022;43(9):2202–11. 10.1038/s41401-021-00839-6. 35042992 10.1038/s41401-021-00839-6 PMC9433456

[113] Li J, Hubisz MJ, Earlie EM, Duran MA, Hong C, Varela AA, et al. Non-cell-autonomous cancer progression from chromosomal instability. Nature. 2023;620(7976):1080–8. 10.1038/s41586-023-06464-z. 37612508 10.1038/s41586-023-06464-z PMC10468402

[114] Guo S, Yao Y, Tang Y, Xin Z, Wu D, Ni C, et al. Radiation-induced tumor immune microenvironments and potential targets for combination therapy. Signal Transduct Target Ther. 2023;8(1):205. 10.1038/s41392-023-01462-z. 37208386 10.1038/s41392-023-01462-z PMC10199044

[115] Wu B, Zhang B, Li B, Wu H, Jiang M. Cold and hot tumors: from molecular mechanisms to targeted therapy. Signal Transduct Target Ther. 2024;9(1):274. 10.1038/s41392-024-01979-x. 39420203 10.1038/s41392-024-01979-x PMC11491057

[116] Wu J, Dobbs N, Yang K, Yan N. Interferon-independent activities of mammalian STING mediate antiviral response and tumor immune evasion. Immunity. 2020;53(1):115-26 e5. 10.1016/j.immuni.2020.06.009. 32640258 10.1016/j.immuni.2020.06.009 PMC7365768

[117] Shen Q, Xu P, Mei C. Role of micronucleus-activated cGAS-STING signaling in antitumor immunity. Zhejiang Da Xue Xue Bao Yi Xue Ban. 2024;53(1):25–34. 10.3724/zdxbyxb-2023-0485. 38273467 10.3724/zdxbyxb-2023-0485 PMC10945493

[118] Cao L, Wang L, Li Z, Wei X, Ding J, Zhou C, et al. Radiotherapy enhances anticancer CD8 T cell responses by cGAMP transfer through LRRC8A/C volume-regulated anion channels. Sci Immunol. 2025;10(108):eadn1630. 10.1126/sciimmunol.adn1630. 40577443 10.1126/sciimmunol.adn1630

[119] Sun Y, Hu H, Liu Z, Xu J, Gao Y, Zhan X, et al. Macrophage STING signaling promotes NK cell to suppress colorectal cancer liver metastasis via 4-1BBL/4-1BB co-stimulation. J Immunother Cancer. 2023. 10.1136/jitc-2022-006481. 36927529 10.1136/jitc-2022-006481 PMC10030919

[120] Lv H, Zong Q, Chen C, Lv G, Xiang W, Xing F, et al. TET2-mediated tumor cGAS triggers endothelial STING activation to regulate vasculature remodeling and anti-tumor immunity in liver cancer. Nat Commun. 2024;15(1):6. 10.1038/s41467-023-43743-9. 38177099 10.1038/s41467-023-43743-9 PMC10766952

[121] Hong C, Schubert M, Tijhuis AE, Requesens M, Roorda M, van den Brink A, et al. cGAS-STING drives the IL-6-dependent survival of chromosomally instable cancers. Nature. 2022;607(7918):366–73. 10.1038/s41586-022-04847-2. 35705809 10.1038/s41586-022-04847-2

[122] Li S, Mirlekar B, Johnson BM, Brickey WJ, Wrobel JA, Yang N, et al. STING-induced regulatory B cells compromise NK function in cancer immunity. Nature. 2022;610(7931):373–80. 10.1038/s41586-022-05254-3. 36198789 10.1038/s41586-022-05254-3 PMC9875944

[123] Li S, Luo M, Wang Z, Feng Q, Wilhelm J, Wang X, et al. Prolonged activation of innate immune pathways by a polyvalent STING agonist. Nat Biomed Eng. 2021;5(5):455–66. 10.1038/s41551-020-00675-9. 33558734 10.1038/s41551-020-00675-9 PMC8126516

[124] Concepcion AR, Wagner LE 2nd, Zhu J, Tao AY, Yang J, Khodadadi-Jamayran A, et al. The volume-regulated anion channel LRRC8C suppresses T cell function by regulating cyclic dinucleotide transport and STING-p53 signaling. Nat Immunol. 2022;23(2):287–302. 10.1038/s41590-021-01105-x. 35105987 10.1038/s41590-021-01105-x PMC8991407

[125] Wang L, Wang FS, Gershwin ME. Human autoimmune diseases: a comprehensive update. J Intern Med. 2015;278(4):369–95. 10.1111/joim.12395. 26212387 10.1111/joim.12395

[126] Song Y, Li J, Wu Y. Evolving understanding of autoimmune mechanisms and new therapeutic strategies of autoimmune disorders. Signal Transduct Target Ther. 2024;9(1):263. 10.1038/s41392-024-01952-8. 39362875 10.1038/s41392-024-01952-8 PMC11452214

[127] Dong M, Fitzgerald KA. DNA-sensing pathways in health, autoinflammatory and autoimmune diseases. Nat Immunol. 2024;25(11):2001–14. 10.1038/s41590-024-01966-y. 39367124 10.1038/s41590-024-01966-y

[128] Kim J, Kim HS, Chung JH. Molecular mechanisms of mitochondrial DNA release and activation of the cGAS-STING pathway. Exp Mol Med. 2023;55(3):510–9. 10.1038/s12276-023-00965-7. 36964253 10.1038/s12276-023-00965-7 PMC10037406

[129] Riley JS, Tait SW. Mitochondrial DNA in inflammation and immunity. EMBO Rep. 2020;21(4):e49799. 10.15252/embr.201949799. 32202065 10.15252/embr.201949799 PMC7132203

[130] Lou H, Pickering MC. Extracellular DNA and autoimmune diseases. Cell Mol Immunol. 2018;15(8):746–55. 10.1038/cmi.2017.136. 29553134 10.1038/cmi.2017.136 PMC6141478

[131] Mondelo-Macia P, Castro-Santos P, Castillo-Garcia A, Muinelo-Romay L, Diaz-Pena R. Circulating free DNA and its emerging role in autoimmune diseases. J Pers Med. 2021. 10.3390/jpm11020151. 33672659 10.3390/jpm11020151 PMC7924199

[132] Hu Y, Chen B, Yang F, Su Y, Yang D, Yao Y, et al. Emerging role of the cGAS-STING signaling pathway in autoimmune diseases: biologic function, mechanisms and clinical prospection. Autoimmun Rev. 2022;21(9):103155. 10.1016/j.autrev.2022.103155. 35902046 10.1016/j.autrev.2022.103155

[133] Gao KM, Marshak-Rothstein A, Fitzgerald KA. Type-1 interferon-dependent and -independent mechanisms in cyclic GMP-AMP synthase-stimulator of interferon genes-driven auto-inflammation. Curr Opin Immunol. 2023;80:102280. 10.1016/j.coi.2022.102280. 36638547 10.1016/j.coi.2022.102280

[134] Jiang J, Zhao M, Chang C, Wu H, Lu Q. Type I interferons in the pathogenesis and treatment of autoimmune diseases. Clin Rev Allergy Immunol. 2020;59(2):248–72. 10.1007/s12016-020-08798-2. 32557263 10.1007/s12016-020-08798-2

[135] Kato Y, Park J, Takamatsu H, Konaka H, Aoki W, Aburaya S, et al. Apoptosis-derived membrane vesicles drive the cGAS-STING pathway and enhance type I IFN production in systemic lupus erythematosus. Ann Rheum Dis. 2018;77(10):1507–15. 10.1136/annrheumdis-2018-212988. 29945921 10.1136/annrheumdis-2018-212988 PMC6161667

[136] Ming X, Yang Z, Huang Y, Wang Z, Zhang Q, Lu C, et al. A chimeric peptide promotes immune surveillance of senescent cells in injury, fibrosis, tumorigenesis and aging. Nat Aging. 2024. 10.1038/s43587-024-00750-9. 39623223 10.1038/s43587-024-00750-9

[137] Di Micco R, Krizhanovsky V, Baker D, d’Adda di Fagagna F. Cellular senescence in ageing: from mechanisms to therapeutic opportunities. Nat Rev Mol Cell Biol. 2021;22(2):75–95. 10.1038/s41580-020-00314-w. 33328614 10.1038/s41580-020-00314-w PMC8344376

[138] Deng Y, Hahn Q, Yu L, Zhu Z, Boyer JA, Wang J, et al. 2’3’-cGAMP interactome identifies 2’3’-cGAMP/Rab18/FosB signaling in cell migration control independent of innate immunity. Sci Adv. 2024;10(42):eado7024. 10.1126/sciadv.ado7024. 39413198 10.1126/sciadv.ado7024 PMC11482326

[139] Khan MS, Khan SU, Khan SU, Suleman M, Shan Ahmad RU, Khan MU, et al. Cardiovascular diseases crossroads: cGAS-STING signaling and disease progression. Curr Probl Cardiol. 2024;49(2):102189. 10.1016/j.cpcardiol.2023.102189. 37956918 10.1016/j.cpcardiol.2023.102189

[140] Abdellatif M, Rainer PP, Sedej S, Kroemer G. Hallmarks of cardiovascular ageing. Nat Rev Cardiol. 2023;20(11):754–77. 10.1038/s41569-023-00881-3. 37193857 10.1038/s41569-023-00881-3

[141] Zhang Q, Shen L, Ruan H, Huang Z. cGAS-STING signaling in cardiovascular diseases. Front Immunol. 2024;15:1402817. 10.3389/fimmu.2024.1402817. 38803502 10.3389/fimmu.2024.1402817 PMC11128581

[142] Wang Y, Deng Y, Chen J, Hahn Q, Umbaugh DS, Zhang Z, et al. cGAS inhibits ALDH2 to suppress lipid droplet function and regulate MASLD progression. Adv Sci (Weinh). 2025. 10.1002/advs.202508576. 41042077 10.1002/advs.202508576 PMC12697864

[143] Pham PT, Fukuda D, Nishimoto S, Kim-Kaneyama JR, Lei XF, Takahashi Y, et al. STING, a cytosolic DNA sensor, plays a critical role in atherogenesis: a link between innate immunity and chronic inflammation caused by lifestyle-related diseases. Eur Heart J. 2021;42(42):4336–48. 10.1093/eurheartj/ehab249. 34226923 10.1093/eurheartj/ehab249

[144] Li X, Chen X, Zheng L, Chen M, Zhang Y, Zhu R, et al. Non-canonical STING-PERK pathway dependent epigenetic regulation of vascular endothelial dysfunction via integrating IRF3 and NF-κB in inflammatory response. Acta Pharm Sin B. 2023;13(12):4765–84. 10.1016/j.apsb.2023.08.015. 38045042 10.1016/j.apsb.2023.08.015 PMC10692388

[145] Cancado de Faria R, Silva L, Teodoro-Castro B, McCommis KS, Shashkova EV, Gonzalo S. A non-canonical cGAS-STING pathway drives cellular and organismal aging. bioRxiv. 2025. 10.1101/2025.04.03.645994. 40638086 10.1073/pnas.2424666122 PMC12280946

[146] Wu J, Wu J, Chen T, Cai J, Ren R. Protein aggregation and its affecting mechanisms in neurodegenerative diseases. Neurochem Int. 2024;180:105880. 10.1016/j.neuint.2024.105880. 39396709 10.1016/j.neuint.2024.105880

[147] Teleanu DM, Niculescu AG, Lungu, II, Radu CI, Vladacenco O, Roza E et al. An Overview of Oxidative Stress, Neuroinflammation, and Neurodegenerative Diseases. Int J Mol Sci. 2022;23(11). 10.3390/ijms23115938. 10.3390/ijms23115938 PMC9180653 35682615

[148] Olufunmilayo EO, Gerke-Duncan MB, Holsinger RMD. Oxidative Stress and Antioxidants in Neurodegenerative Disorders. Antioxidants (Basel). 2023;12(2). 10.3390/antiox12020517. 10.3390/antiox12020517 PMC9952099 36830075

[149] Paul BD, Snyder SH, Bohr VA. Signaling by cGAS-STING in neurodegeneration, neuroinflammation, and aging. Trends Neurosci. 2021;44(2):83–96. 10.1016/j.tins.2020.10.008. 33187730 10.1016/j.tins.2020.10.008 PMC8662531

[150] Chang H, Li Z, Zhang W, Lin C, Shen Y, Zhang G, et al. Transfer of cGAMP from neuron to microglia activates microglial type I interferon responses after subarachnoid hemorrhage. Cell Commun Signal. 2024;22(1):3. 10.1186/s12964-023-01362-3. 38169382 10.1186/s12964-023-01362-3 PMC10763285

[151] Wang Y, Niu W, Zhu S, Sun J, Lv J, Wang N, et al. STING agonist cGAMP attenuates sleep deprivation-induced neuroinflammation and cognitive deficits via TREM2 up-regulation. Inflammation. 2024;47(6):2129–44. 10.1007/s10753-024-02029-y. 38668837 10.1007/s10753-024-02029-y

[152] Pesaresi M, Sebastian-Perez R, Cosma MP. Dedifferentiation, transdifferentiation and cell fusion: in vivo reprogramming strategies for regenerative medicine. FEBS J. 2019;286(6):1074–93. 10.1111/febs.14633. 30103260 10.1111/febs.14633

[153] Terada N, Hamazaki T, Oka M, Hoki M, Mastalerz DM, Nakano Y, et al. Bone marrow cells adopt the phenotype of other cells by spontaneous cell fusion. Nature. 2002;416(6880):542–5. 10.1038/nature730. 11932747 10.1038/nature730

[154] Lopez-Otin C, Blasco MA, Partridge L, Serrano M, Kroemer G. Hallmarks of aging: an expanding universe. Cell. 2023;186(2):243–78. 10.1016/j.cell.2022.11.001. 36599349 10.1016/j.cell.2022.11.001

[155] Schumacher B, Pothof J, Vijg J, Hoeijmakers JHJ. The central role of DNA damage in the ageing process. Nature. 2021;592(7856):695–703. 10.1038/s41586-021-03307-7. 33911272 10.1038/s41586-021-03307-7 PMC9844150

[156] Miller KN, Li B, Pierce-Hoffman HR, Patel S, Lei X, Rajesh A, et al. p53 enhances DNA repair and suppresses cytoplasmic chromatin fragments and inflammation in senescent cells. Nat Commun. 2025;16(1):2229. 10.1038/s41467-025-57229-3. 40044657 10.1038/s41467-025-57229-3 PMC11882782

[157] Li T, Chen ZJ. The cGAS-cGAMP-STING pathway connects DNA damage to inflammation, senescence, and cancer. J Exp Med. 2018;215(5):1287–99. 10.1084/jem.20180139. 29622565 10.1084/jem.20180139 PMC5940270

[158] Yu Q, Katlinskaya YV, Carbone CJ, Zhao B, Katlinski KV, Zheng H, et al. DNA-damage-induced type I interferon promotes senescence and inhibits stem cell function. Cell Rep. 2015;11(5):785–97. 10.1016/j.celrep.2015.03.069. 25921537 10.1016/j.celrep.2015.03.069 PMC4426031

[159] Banerjee D, Langberg K, Abbas S, Odermatt E, Yerramothu P, Volaric M, et al. A non-canonical, interferon-independent signaling activity of cGAMP triggers DNA damage response signaling. Nat Commun. 2021;12(1):6207. 10.1038/s41467-021-26240-9. 34707113 10.1038/s41467-021-26240-9 PMC8551335

[160] Duan Y, Du A, Gu J, Duan G, Wang C, Gui X, et al. PARylation regulates stress granule dynamics, phase separation, and neurotoxicity of disease-related RNA-binding proteins. Cell Res. 2019;29(3):233–47. 10.1038/s41422-019-0141-z. 30728452 10.1038/s41422-019-0141-z PMC6460439

[161] Lee JH, Hussain M, Kim EW, Cheng SJ, Leung AKL, Fakouri NB, et al. Mitochondrial PARP1 regulates NAD(+)-dependent poly ADP-ribosylation of mitochondrial nucleoids. Exp Mol Med. 2022;54(12):2135–47. 10.1038/s12276-022-00894-x. 36473936 10.1038/s12276-022-00894-x PMC9794712

[162] Covarrubias AJ, Perrone R, Grozio A, Verdin E. NAD(+) metabolism and its roles in cellular processes during ageing. Nat Rev Mol Cell Biol. 2021;22(2):119–41. 10.1038/s41580-020-00313-x. 33353981 10.1038/s41580-020-00313-x PMC7963035

[163] Covarrubias AJ, Kale A, Perrone R, Lopez-Dominguez JA, Pisco AO, Kasler HG, et al. Senescent cells promote tissue NAD(+) decline during ageing via the activation of CD38(+) macrophages. Nat Metab. 2020;2(11):1265–83. 10.1038/s42255-020-00305-3. 33199924 10.1038/s42255-020-00305-3 PMC7908681

[164] Li X, Li C, Zhang W, Wang Y, Qian P, Huang H. Inflammation and aging: signaling pathways and intervention therapies. Signal Transduct Target Ther. 2023;8(1):239. 10.1038/s41392-023-01502-8. 37291105 10.1038/s41392-023-01502-8 PMC10248351

[165] Teissier T, Boulanger E, Cox LS. Interconnections between Inflammageing and Immunosenescence during Ageing. Cells. 2022;11(3). 10.3390/cells11030359. 10.3390/cells11030359 PMC8834134 35159168

[166] Bleve A, Motta F, Durante B, Pandolfo C, Selmi C, Sica A. Immunosenescence, inflammaging, and frailty: role of myeloid cells in age-related diseases. Clin Rev Allergy Immunol. 2023;64(2):123–44. 10.1007/s12016-021-08909-7. 35031957 10.1007/s12016-021-08909-7 PMC8760106

[167] Lopes-Paciencia S, Saint-Germain E, Rowell MC, Ruiz AF, Kalegari P, Ferbeyre G. The senescence-associated secretory phenotype and its regulation. Cytokine. 2019;117:15–22. 10.1016/j.cyto.2019.01.013. 30776684 10.1016/j.cyto.2019.01.013

[168] Han X, Lei Q, Xie J, Liu H, Li J, Zhang X, et al. Potential regulators of the senescence-associated secretory phenotype during senescence and aging. J Gerontol A Biol Sci Med Sci. 2022;77(11):2207–18. 10.1093/gerona/glac097. 35524726 10.1093/gerona/glac097

[169] Decout A, Katz JD, Venkatraman S, Ablasser A. The cGAS-STING pathway as a therapeutic target in inflammatory diseases. Nat Rev Immunol. 2021;21(9):548–69. 10.1038/s41577-021-00524-z. 33833439 10.1038/s41577-021-00524-z PMC8029610

[170] Chen MS, Lee RT, Garbern JC. Senescence mechanisms and targets in the heart. Cardiovasc Res. 2022;118(5):1173–87. 10.1093/cvr/cvab161. 33963378 10.1093/cvr/cvab161 PMC8953446

[171] Ohtani N. The roles and mechanisms of senescence-associated secretory phenotype (SASP): can it be controlled by senolysis? Inflamm Regen. 2022;42(1):11. 10.1186/s41232-022-00197-8. 35365245 10.1186/s41232-022-00197-8 PMC8976373

[172] Swanson KV, Junkins RD, Kurkjian CJ, Holley-Guthrie E, Pendse AA, El Morabiti R, et al. A noncanonical function of cGAMP in inflammasome priming and activation. J Exp Med. 2017;214(12):3611–26. 10.1084/jem.20171749. 29030458 10.1084/jem.20171749 PMC5716045

[173] Muela-Zarzuela I, Suarez-Rivero JM, Gallardo-Orihuela A, Wang C, Izawa K, de Gregorio-Procopio M, et al. NLRP1 inflammasome promotes senescence and senescence-associated secretory phenotype. Inflamm Res. 2024;73(8):1253–66. 10.1007/s00011-024-01892-7. 38907167 10.1007/s00011-024-01892-7 PMC11281979

[174] Sharma BR, Kanneganti TD. NLRP3 inflammasome in cancer and metabolic diseases. Nat Immunol. 2021;22(5):550–9. 10.1038/s41590-021-00886-5. 33707781 10.1038/s41590-021-00886-5 PMC8132572

[175] Dossou AS, Basu A. The Emerging Roles of mTORC1 in Macromanaging Autophagy. Cancers (Basel). 2019;11(10). 10.3390/cancers11101422. 10.3390/cancers11101422 PMC6826502 31554253

[176] Panwar V, Singh A, Bhatt M, Tonk RK, Azizov S, Raza AS, et al. Multifaceted role of mTOR (mammalian target of rapamycin) signaling pathway in human health and disease. Signal Transduct Target Ther. 2023;8(1):375. 10.1038/s41392-023-01608-z. 37779156 10.1038/s41392-023-01608-z PMC10543444

[177] Imanishi T, Unno M, Kobayashi W, Yoneda N, Matsuda S, Ikeda K et al. Reciprocal regulation of STING and TCR signaling by mTORC1 for T-cell activation and function. Life Sci Alliance. 2019;2(1). 10.26508/lsa.201800282. 10.26508/lsa.201800282 PMC6348487 30683688

[178] Gui X, Yang H, Li T, Tan X, Shi P, Li M, et al. Autophagy induction via STING trafficking is a primordial function of the cGAS pathway. Nature. 2019;567(7747):262–6. 10.1038/s41586-019-1006-9. 30842662 10.1038/s41586-019-1006-9 PMC9417302

[179] Pan M, Yin Y, Hu T, Wang X, Jia T, Sun J, et al. UXT attenuates the CGAS-STING1 signaling by targeting STING1 for autophagic degradation. Autophagy. 2023;19(2):440–56. 10.1080/15548627.2022.2076192. 35543189 10.1080/15548627.2022.2076192 PMC9851252

[180] Xiong Y, Tang YD, Zheng C. The crosstalk between the caspase family and the cGAS‒STING signaling pathway. J Mol Cell Biol. 2021;13(10):739–47. 10.1093/jmcb/mjab071. 34718659 10.1093/jmcb/mjab071 PMC8718194

[181] Zierhut C, Yamaguchi N, Paredes M, Luo JD, Carroll T, Funabiki H. The Cytoplasmic DNA Sensor cGAS Promotes Mitotic Cell Death. Cell. 2019;178(2):302–15 e23. 10.1016/j.cell.2019.05.035. 10.1016/j.cell.2019.05.035 PMC6693521 31299200

[182] Sorice M. Crosstalk of Autophagy and Apoptosis. Cells. 2022;11(9). 10.3390/cells11091479. 10.3390/cells11091479 PMC9102887 35563785

[183] Xu Y, Chen C, Liao Z, Xu P. cGAS-STING signaling in cell death: mechanisms of action and implications in pathologies. Eur J Immunol. 2023;53(9):e2350386. 10.1002/eji.202350386. 37424054 10.1002/eji.202350386

[184] Li XD, Wu J, Gao D, Wang H, Sun L, Chen ZJ. Pivotal roles of cGAS-cGAMP signaling in antiviral defense and immune adjuvant effects. Science. 2013;341(6152):1390–4. 10.1126/science.1244040. 23989956 10.1126/science.1244040 PMC3863637

[185] Zhu W, Wei L, Dong C, Wang Y, Kim J, Ma Y, et al. cGAMP-adjuvanted multivalent influenza mRNA vaccines induce broadly protective immunity through cutaneous vaccination in mice. Mol Ther Nucleic Acids. 2022;30:421–37. 10.1016/j.omtn.2022.10.024. 36420215 10.1016/j.omtn.2022.10.024 PMC9668623

[186] Leekha A, Saeedi A, Kumar M, Sefat K, Martinez-Paniagua M, Meng H, et al. An intranasal nanoparticle STING agonist protects against respiratory viruses in animal models. Nat Commun. 2024;15(1):6053. 10.1038/s41467-024-50234-y. 39025863 10.1038/s41467-024-50234-y PMC11258242

[187] Wang J, Li P, Yu Y, Fu Y, Jiang H, Lu M et al. Pulmonary surfactant-biomimetic nanoparticles potentiate heterosubtypic influenza immunity. Science. 2020;367(6480). 10.1126/science.aau0810. 10.1126/science.aau0810 PMC7432993 32079747

[188] Sefat K, Kumar M, Kehl S, Kulkarni R, Leekha A, Paniagua MM, et al. An intranasal nanoparticle vaccine elicits protective immunity against *Mycobacterium tuberculosis*. Vaccine. 2024;42(22):125909. 10.1016/j.vaccine.2024.04.055. 38704256 10.1016/j.vaccine.2024.04.055 PMC12279440

[189] Batty CJ, Gallovic MD, Williams J, Ross TM, Bachelder EM, Ainslie KM. Multiplexed electrospray enables high throughput production of cGAMP microparticles to serve as an adjuvant for a broadly acting influenza vaccine. Int J Pharm. 2022;622:121839. 10.1016/j.ijpharm.2022.121839. 35623484 10.1016/j.ijpharm.2022.121839 PMC9484837

[190] A viral RNA molecule activates the bacterial immune system during infection. Nature. 2023. 10.1038/d41586-023-03364-0. 10.1038/d41586-023-03364-0 37968462

[191] Xu X, Wang X, Liao YP, Luo L, Nel AE. Reprogramming the tolerogenic immune response against pancreatic cancer metastases by lipid nanoparticles delivering a STING agonist plus mutant KRAS mRNA. ACS Nano. 2025;19(9):8579–94. 10.1021/acsnano.4c14102. 40025875 10.1021/acsnano.4c14102 PMC11912578

[192] Li T, Cheng H, Yuan H, Xu Q, Shu C, Zhang Y, et al. Antitumor activity of cGAMP via stimulation of cGAS-cGAMP-STING-IRF3 mediated innate immune response. Sci Rep. 2016;6:19049. 10.1038/srep19049. 26754564 10.1038/srep19049 PMC4709567

[193] Chen M, Lei S, Zhou Z, Wang M, Feng C, Gao X, et al. Design, synthesis, and pharmacological evaluation of Spiro[carbazole-3,3’-pyrrolidine] derivatives as cGAS inhibitors for treatment of acute lung injury. J Med Chem. 2024;67(8):6268–91. 10.1021/acs.jmedchem.3c02229. 38619191 10.1021/acs.jmedchem.3c02229

[194] Qu J, Cai Y, Li F, Li X, Liu R. Potential therapeutic strategies for colitis and colon cancer: bidirectional targeting STING pathway. EBioMedicine. 2025;111:105491. 10.1016/j.ebiom.2024.105491. 39644772 10.1016/j.ebiom.2024.105491 PMC11665664

[195] Zhang Z, Jiang J, Wu G, Wei X, Weng Y, Huang LS. The cGAS-STING Pathway in Pulmonary Diseases: Mechanisms and Therapeutic Potential. Int J Mol Sci. 2025;26(21). 10.3390/ijms262110423. 10.3390/ijms262110423 PMC12607373 41226461

[196] Skeldon AM, Wang L, Sgarioto N, Beveridge RE, Chan S, Dorich S, et al. Structural insight into the cGAS active site explains differences between therapeutically relevant species. Commun Chem. 2025;8(1):88. 10.1038/s42004-025-01481-7. 40121343 10.1038/s42004-025-01481-7 PMC11929900

[197] Li H, Liu C, Li R, Zhou L, Ran Y, Yang Q, et al. AARS1 and AARS2 sense L-lactate to regulate cGAS as global lysine lactyltransferases. Nature. 2024;634(8036):1229–37. 10.1038/s41586-024-07992-y. 39322678 10.1038/s41586-024-07992-y

[198] Seok JK, Kim M, Kang HC, Cho YY, Lee HS, Lee JY. Beyond DNA sensing: expanding the role of cGAS/STING in immunity and diseases. Arch Pharm Res. 2023;46(6):500–34. 10.1007/s12272-023-01452-3. 37354378 10.1007/s12272-023-01452-3 PMC10333371

[199] Goswami A, Goyal S, Khurana P, Singh K, Deb B, Kulkarni A. Small molecule innate immune modulators in cancer therapy. Front Immunol. 2024;15:1395655. 10.3389/fimmu.2024.1395655. 39318624 10.3389/fimmu.2024.1395655 PMC11419979

[200] Onyedibe KI, Wang M, Sintim HO. ENPP1, an old enzyme with new functions, and small molecule inhibitors-a STING in the tale of ENPP1. Molecules. 2019. 10.3390/molecules24224192. 31752288 10.3390/molecules24224192 PMC6891441

[201] Wang S, Bohnert V, Joseph AJ, Sudaryo V, Skariah G, Swinderman JT, et al. ENPP1 is an innate immune checkpoint of the anticancer cGAMP-STING pathway in breast cancer. Proc Natl Acad Sci U S A. 2023;120(52):e2313693120. 10.1073/pnas.2313693120. 38117852 10.1073/pnas.2313693120 PMC10756298

[202] Stagg J, Golden E, Wennerberg E, Demaria S. The interplay between the DNA damage response and ectonucleotidases modulates tumor response to therapy. Sci Immunol. 2023;8(85):eabq3015. 10.1126/sciimmunol.abq3015. 37418547 10.1126/sciimmunol.abq3015 PMC10394739

[203] Yan Y, Tan X, Song B, Yi M, Chu Q, Wu K. Breaking barriers: the cGAS-STING pathway as a novel frontier in cancer immunotherapy. Cancer Commun (Lond). 2025;45(11):1513–46. 10.1002/cac2.70067. 41077675 10.1002/cac2.70067 PMC12629869

[204] Vennard CS, Oladeji SM, Sintim HO. Inhibitors of cyclic dinucleotide phosphodiesterases and cyclic oligonucleotide ring nucleases as potential drugs for various diseases. Cells. 2025. 10.3390/cells14090663. 40358186 10.3390/cells14090663 PMC12072042

[205] Zeng Q, Liu M, Wang Z, Zhou R, Ai K. Enhancing radiotherapy-induced anti-tumor immunity via nanoparticle-mediated STING agonist synergy. Mol Cancer. 2025;24(1):176. 10.1186/s12943-025-02366-y. 40500702 10.1186/s12943-025-02366-y PMC12153120

[206] Fahey CG, Cordova AF, Gedeon PC, Barbie DA. Targeting STING to generate therapeutic anti-tumor immunity. Cancer Cell. 2026;44(2):260–80. 10.1016/j.ccell.2025.12.002. 41448179 10.1016/j.ccell.2025.12.002 PMC12779296

[207] Huang N, Liu Z, Lei H, Liu X. The roles of the mtDNA-cGAS-STING axis in tumor immunity: from immune activation to immune evasion. Front Immunol. 2025;16:1739559. 10.3389/fimmu.2025.1739559. 41601699 10.3389/fimmu.2025.1739559 PMC12833221

[208] Pu C, Cui H, Yu H, Cheng X, Zhang M, Qin L, et al. Oral ENPP1 inhibitor designed using generative AI as next generation STING modulator for solid tumors. Nat Commun. 2025;16(1):4793. 10.1038/s41467-025-59874-0. 40410143 10.1038/s41467-025-59874-0 PMC12102218

[209] Yang R, Wang B, Su Z, Song Y, Zhang Y, Liu Y, et al. Methotrexate exerts antitumor immune activity and improves the clinical efficacy of immunotherapy in patients with solid tumors. Sci Transl Med. 2025;17(801):eadn6921. 10.1126/scitranslmed.adn6921. 40465690 10.1126/scitranslmed.adn6921

[210] Figgins EL, Arora P, Gao D, Porcelli E, Ahmed R, Daep CA, et al. Enhancement of innate immunity in gingival epithelial cells by vitamin D and HDAC inhibitors. Front Oral Health. 2024;5:1378566. 10.3389/froh.2024.1378566. 38567313 10.3389/froh.2024.1378566 PMC10986367

[211] Tokajuk J, Deptula P, Piktel E, Daniluk T, Chmielewska S, Wollny T, et al. Cathelicidin LL-37 in health and diseases of the oral cavity. Biomedicines. 2022. 10.3390/biomedicines10051086. 35625823 10.3390/biomedicines10051086 PMC9138798

[212] McAndrews KM, Che SPY, LeBleu VS, Kalluri R. Effective delivery of STING agonist using exosomes suppresses tumor growth and enhances antitumor immunity. J Biol Chem. 2021;296:100523. 10.1016/j.jbc.2021.100523. 33711340 10.1016/j.jbc.2021.100523 PMC8042450

[213] Hao Y, Ji Z, Zhou H, Wu D, Gu Z, Wang D, et al. Lipid-based nanoparticles as drug delivery systems for cancer immunotherapy. MedComm. 2023;4(4):e339. 10.1002/mco2.339. 37560754 10.1002/mco2.339 PMC10407046

[214] Nerdinger YG, Binder AK, Bremm F, Feuchter N, Schaft N, Dorrie J. STINGing cancer: development, clinical application, and targeted delivery of STING agonists. Int J Mol Sci. 2025. 10.3390/ijms26189008. 41009575 10.3390/ijms26189008 PMC12470042

[215] Chauveau L, Bridgeman A, Tan TK, Beveridge R, Frost JN, Rijal P, et al. Inclusion of cGAMP within virus-like particle vaccines enhances their immunogenicity. EMBO Rep. 2021;22(8):e52447. 10.15252/embr.202152447. 34142428 10.15252/embr.202152447 PMC8339669

[216] Watkins-Schulz R, Batty CJ, Stiepel RT, Schmidt ME, Sandor AM, Chou WC, et al. Microparticle delivery of a STING agonist enables indirect activation of NK cells by antigen-presenting cells. Mol Pharm. 2022;19(9):3125–38. 10.1021/acs.molpharmaceut.2c00207. 35913984 10.1021/acs.molpharmaceut.2c00207 PMC13335844

[217] Pena ES, Batty CJ, Hendy DA, Yang S, Ontiveros-Padilla L, Stiepel RT, et al. Comparative study of acetalated-dextran microparticle fabrication methods for a clinically translatable subunit-based influenza vaccine. Int J Pharm. 2024;652:123836. 10.1016/j.ijpharm.2024.123836. 38266940 10.1016/j.ijpharm.2024.123836 PMC10923012

[218] Junkins RD, Gallovic MD, Johnson BM, Collier MA, Watkins-Schulz R, Cheng N, et al. A robust microparticle platform for a STING-targeted adjuvant that enhances both humoral and cellular immunity during vaccination. J Control Release. 2018;270:1–13. 10.1016/j.jconrel.2017.11.030. 29170142 10.1016/j.jconrel.2017.11.030 PMC5808851

[219] Chen C, Tong Y, Zheng Y, Shi Y, Chen Z, Li J, et al. Cytosolic delivery of thiolated Mn-cGAMP nanovaccine to enhance the antitumor immune responses. Small. 2021;17(17):e2006970. 10.1002/smll.202006970. 33719177 10.1002/smll.202006970

[220] Chen X, Xu Z, Li T, Thakur A, Wen Y, Zhang K, et al. Nanomaterial-encapsulated STING agonists for immune modulation in cancer therapy. Biomark Res. 2024;12(1):2. 10.1186/s40364-023-00551-z. 38185685 10.1186/s40364-023-00551-z PMC10773049

[221] Yuan P, Yan X, Zong X, Li X, Yang C, Chen X, et al. Modulating Elasticity of Liposome for Enhanced Cancer Immunotherapy. ACS Nano. 2024;18(34):23797–811. 10.1021/acsnano.4c09094. 39140567 10.1021/acsnano.4c09094

[222] An Y, Zhu J, Xie Q, Feng J, Gong Y, Fan Q, et al. Tumor exosomal ENPP1 hydrolyzes cGAMP to inhibit cGAS-STING signaling. Adv Sci (Weinh). 2024;11(20):e2308131. 10.1002/advs.202308131. 38498770 10.1002/advs.202308131 PMC11132070

[223] Borza R, Salgado-Polo F, Moolenaar WH, Perrakis A. Structure and function of the ecto-nucleotide pyrophosphatase/phosphodiesterase (ENPP) family: tidying up diversity. J Biol Chem. 2022;298(2):101526. 10.1016/j.jbc.2021.101526. 34958798 10.1016/j.jbc.2021.101526 PMC8808174

[224] Watanabe R, Fujita N, Sato Y, Kobayashi T, Morita M, Oike T, et al. Enpp1 is an anti-aging factor that regulates Klotho under phosphate overload conditions. Sci Rep. 2017;7(1):7786. 10.1038/s41598-017-07341-2. 28798354 10.1038/s41598-017-07341-2 PMC5552841

[225] Carozza JA, Cordova AF, Brown JA, AlSaif Y, Bohnert V, Cao X, et al. ENPP1’s regulation of extracellular cGAMP is a ubiquitous mechanism of attenuating STING signaling. Proc Natl Acad Sci U S A. 2022;119(21):e2119189119. 10.1073/pnas.2119189119. 35588451 10.1073/pnas.2119189119 PMC9173814

[226] Wang Z, Hou Y, Liu P, Wu R, Yang J, Fan S, et al. Membrane integrity changes upon viral infection activate sphingomyelinase SMPDL3B to restrict cGAS-STING signaling via cGAMP degradation. Immunity. 2025;58(11):2670-84 e10. 10.1016/j.immuni.2025.10.007. 41175872 10.1016/j.immuni.2025.10.007

[227] Meng W, He C, Hao Y, Wang L, Li L, Zhu G. Prospects and challenges of extracellular vesicle-based drug delivery system: considering cell source. Drug Deliv. 2020;27(1):585–98. 10.1080/10717544.2020.1748758. 32264719 10.1080/10717544.2020.1748758 PMC7178886

[228] Kim HI, Park J, Zhu Y, Wang X, Han Y, Zhang D. Recent advances in extracellular vesicles for therapeutic cargo delivery. Exp Mol Med. 2024;56(4):836–49. 10.1038/s12276-024-01201-6. 38556545 10.1038/s12276-024-01201-6 PMC11059217

[229] Manno M, Bongiovanni A, Margolis L, Bergese P, Arosio P. The physico-chemical landscape of extracellular vesicles. Nat Rev Bioeng. 2024. 10.1038/s44222-024-00255-5.

[230] Zhong XF, Sun X. Nanomedicines based on nanoscale metal-organic frameworks for cancer immunotherapy. Acta Pharmacol Sin. 2020;41(7):928–35. 10.1038/s41401-020-0414-6. 32355277 10.1038/s41401-020-0414-6 PMC7468577

[231] Liu Y, Pu F. Updated roles of cGAS-STING signaling in autoimmune diseases. Front Immunol. 2023;14:1254915. 10.3389/fimmu.2023.1254915. 37781360 10.3389/fimmu.2023.1254915 PMC10538533

[232] Su CI, Kao YT, Chang CC, Chang Y, Ho TS, Sun HS, et al. DNA-induced 2’3’-cGAMP enhances haplotype-specific human STING cleavage by dengue protease. Proc Natl Acad Sci U S A. 2020;117(27):15947–54. 10.1073/pnas.1922243117. 32576686 10.1073/pnas.1922243117 PMC7354927

[233] Gumanova NG, Bogdanova NL, Gorshkov AY. Associations of serum levels of cGAMP in the context of COVID-19 infection, atherosclerosis, sterile inflammation, and functional endothelial biomarkers in patients with coronary heart disease and healthy volunteers. Horm Mol Biol Clin Investig. 2025;46(2):93–101. 10.1515/hmbci-2024-0073. 39898866 10.1515/hmbci-2024-0073

[234] Ji S, Xiong M, Chen H, Liu Y, Zhou L, Hong Y, et al. Cellular rejuvenation: molecular mechanisms and potential therapeutic interventions for diseases. Signal Transduct Target Ther. 2023;8(1):116. 10.1038/s41392-023-01343-5. 36918530 10.1038/s41392-023-01343-5 PMC10015098

[235] Vaiserman A, De Falco E, Koliada A, Maslova O, Balistreri CR. Anti-ageing gene therapy: Not so far away? Ageing Res Rev. 2019;56:100977. 10.1016/j.arr.2019.100977. 31669577 10.1016/j.arr.2019.100977

[236] Yu J, Li T, Zhu J. Gene Therapy Strategies Targeting Aging-Related Diseases. Aging Dis. 2023;14(2):398–417. 10.14336/AD.2022.00725. 37008065 10.14336/AD.2022.00725 PMC10017145

[237] Yue B, Gao W, Lovell JF, Jin H, Huang J. The cGAS-STING pathway in cancer immunity: dual roles, therapeutic strategies, and clinical challenges. Essays Biochem. 2025. 10.1042/EBC20253006. 40052963 10.1042/EBC20253006 PMC12204001

[238] Yu T, Fleishman JS, Wang H, Liu X, Huo L. cGAS-STING targeting offers novel therapeutic regimen in sepsis-associated organ dysfunction. Cell Biol Toxicol. 2025;41(1):113. 10.1007/s10565-025-10051-5. 40608126 10.1007/s10565-025-10051-5 PMC12226679

[239] Chen S, Li J, Yan L, Zhang X, Huang J, Zhou P. Electroacupuncture alleviates the symptom of depression in mice by regulating the cGAS-STING-NLRP3 signaling. Aging (Albany NY). 2024;16(8):6731–44. 10.18632/aging.205596. 38643466 10.18632/aging.205596 PMC11087112

[240] Petrovic M, Borchard G, Jordan O. Considerations for the delivery of STING ligands in cancer immunotherapy. J Control Release. 2021;339:235–47. 10.1016/j.jconrel.2021.09.033. 34592386 10.1016/j.jconrel.2021.09.033

[241] Zhu T, Xiao Y, Chen Z, Ding H, Chen S, Jiang G, et al. Inhalable nanovesicles loaded with a STING agonist enhance CAR-T cell activity against solid tumors in the lung. Nat Commun. 2025;16(1):262. 10.1038/s41467-024-55751-4. 39747173 10.1038/s41467-024-55751-4 PMC11695690

[242] Zhou Z, Liu H, Ye M. Research progress on the nucleoside/nucleotide-loaded nanomedicines. Zhejiang Da Xue Xue Bao Yi Xue Ban. 2023;52(3):279–84. 10.3724/zdxbyxb-2022-0701. 37476939 10.3724/zdxbyxb-2022-0701 PMC10409901

[243] Jain S, Kumar M, Kumar P, Verma J, Rosenholm JM, Bansal KK, et al. Lipid-polymer hybrid nanosystems: a rational fusion for advanced therapeutic delivery. J Funct Biomater. 2023. 10.3390/jfb14090437. 37754852 10.3390/jfb14090437 PMC10531762

[244] Smarduch S, Moreno-Velasquez SD, Ilic D, Dadsena S, Morant R, Ciprinidis A, et al. A novel biosensor for the spatiotemporal analysis of STING activation during innate immune responses to dsDNA. EMBO J. 2025;44(7):2157–82. 10.1038/s44318-025-00370-y. 39984755 10.1038/s44318-025-00370-y PMC11962129

[245] Lebrec V, Afshar N, Davies LR, Kujirai T, Kanellou A, Tidu F et al. A microscopy reporter for cGAMP reveals rare cGAS activation following DNA damage, and a lack of correlation with micronuclear cGAS enrichment. 2024:2024.05.13.593978. 10.1101/2024.05.13.593978 %J bioRxiv.

